# Bioactive Compounds and In Vitro Antibacterial Activity of Hemp Leaves and Inflorescences as Potential Functional Feed Ingredients for Canine Nutrition

**DOI:** 10.3390/ani16172635

**Published:** 2026-08-22

**Authors:** Weronika Jacuńska, Wioletta Biel, Piotr Waligórski, Robert Witkowicz, Sławomir Zych

**Affiliations:** 1 Department of Monogastric Animal Sciences, Division of Animal Nutrition and Food, Faculty of Biotechnology and Animal Science, West Pomeranian University of Technology in Szczecin, 29 Klemensa Janickiego Street, 71-270 Szczecin, Poland; wioletta.biel@zut.edu.pl; 2 The Franciszek Górski Institute of Plant Physiology Polish Academy of Sciences, 21 Niezapominajek Street, 30-239 Kraków, Poland; pewalig7@gmail.com; 3 Department of Agroecology and Crop Production, Faculty of Agriculture and Economics, University of Agriculture in Kraków, 21 Mickiewicza Street, 31-120 Kraków, Poland; robert.witkowicz@urk.edu.pl; 4 Department of Microbiology and Biotechnology, Faculty of Biotechnology and Animal Science, West Pomeranian University of Technology in Szczecin, 45 Piastów Avenue, 70-310 Szczecin, Poland; slawomir.zych@zut.edu.pl

**Keywords:** biomass valorization, *Cannabis sativa* L., cannabinoids, dog nutrition, functional feed ingredients, industrial hemp, natural antimicrobials, phytochemical analysis, terpenes, time–kill assay

## Abstract

Hemp (*Cannabis sativa* L.) inflorescences have been extensively characterized for their bioactive compounds, whereas less information is available on hemp leaves despite their potential as a source of nutrients and phytochemicals. This study compared leaves and inflorescences of four organically grown hemp cultivars (Finola, Futura 75, Dioica, and Kompolti) in terms of cannabinoid, terpene, and fatty acid profiles, lipid quality indices, and in vitro antibacterial activity. Antibacterial activity against *Staphylococcus aureus*, *Escherichia coli*, and *Pseudomonas aeruginosa* was evaluated using MIC, MBC, and time–kill assays. Total cannabinoid content was approximately 2.7-fold higher in inflorescences than in leaves, which were also generally richer in terpenes. Leaves showed a more favorable fatty acid profile, with higher n-3 polyunsaturated fatty acid proportions and a lower n-6/n-3 ratio. All extracts exhibited antibacterial activity. Among the tested bacteria, *S. aureus* was the most susceptible to the hemp extracts, with both growth inhibition and bactericidal activity observed at the lowest tested concentration (MIC/MBC = 0.005%), compared with 0.625% for *E. coli* and 1.25–2.5% for *P. aeruginosa*, depending on the extract. Finola and Kompolti inflorescence extracts showed the fastest bactericidal activity against *S. aureus.* These findings demonstrate distinct and complementary characteristics of hemp leaves and inflorescences and provide a basis for further investigation of their potential relevance to canine nutrition.

## Simple Summary

The pet food industry is increasingly looking for natural ingredients that can improve both the nutritional value and microbiological safety of dog diets while making better use of agricultural resources. Industrial hemp cultivation generates large amounts of leaves that are often underutilized, although they contain valuable natural compounds. This study compared hemp leaves and inflorescences from four industrial hemp cultivars to determine whether these plant materials could be useful in canine nutrition. The results showed that leaves provide a favorable fatty acid profile, whereas inflorescences are richer in naturally occurring plant compounds such as cannabinoids and terpenes. Extracts from both plant parts also inhibited the growth of bacteria under laboratory conditions. These findings suggest that different hemp plant parts may have complementary roles as functional ingredients in dog foods while supporting more sustainable use of hemp biomass. Further studies in complete diets and feeding trials are needed before practical application.

## 1. Introduction

Limited opportunities to expand agricultural production and cultivated land, together with the continuously increasing demand for food and feed driven by population growth, have highlighted the need for more efficient utilization of plant biomass resources [1,2,3,4]. One approach is the valorization of plant parts that have traditionally remained underutilized or have had only limited applications, despite being valuable sources of nutrients and bioactive compounds [5,6]. This approach supports the principles of the circular bioeconomy and creates opportunities for developing novel feed ingredients, including ingredients for companion animals that provide nutritional value together with potentially health-promoting compounds [1,7,8]. Industrial hemp (*Cannabis sativa* L.) is among the plant species with considerable potential, and its utilization need not be limited to inflorescences, seeds, and the oil extracted from them. Hemp leaves and inflorescences contain numerous nutrients and bioactive compounds that may be of interest for the development of pet food ingredients, as has already been demonstrated for foods intended for human consumption [9,10,11,12,13]. Inflorescences are the primary site of cannabinoid biosynthesis and accumulation [14,15,16,17]. However, several studies have shown that, in terms of antioxidant and anti-inflammatory activity, leaves may equal or even surpass inflorescences for selected parameters [18,19,20]. Therefore, evaluating their chemical composition and comparing it with that of inflorescences may provide valuable insights into the properties of these hemp-derived raw materials relevant to their potential future use in animal nutrition. However, their suitability as functional feed ingredients cannot be established from compositional and in vitro analyses alone and requires further evaluation of digestibility, palatability, bioavailability, safety, and feeding efficacy [21,22,23,24,25,26].

More than 600 chemical compounds have been identified in *Cannabis sativa*, including cannabinoids, terpenes, flavonoids, and lipids [14,27,28,29,30,31,32]. Among these, cannabinoids have attracted the greatest scientific interest as the most characteristic group of compounds found in hemp. In industrial hemp cultivars authorized for cultivation within the European Union, the total Δ9-tetrahydrocannabinol (Δ9-THC) content, calculated as the sum of Δ9-THC and its precursor tetrahydrocannabinolic acid (THC-A), must not exceed 0.3% on a dry matter basis [33].

Hemp seed oil is characterized by a high content of unsaturated fatty acids (UFA), with polyunsaturated fatty acids (PUFA) accounting for up to 90% of the total fatty acid fraction [10,34]. Linoleic acid (LA, n-6) and α-linolenic acid (ALA, n-3) are the predominant fatty acids [35,36]. Hemp oil is also distinguished by an n-6/n-3 fatty acid ratio of approximately 3:1, which is considered nutritionally favorable [37,38]. In companion animals, these fatty acids contribute to maintaining skin barrier integrity and healthy skin and coat. Therefore, an adequate supply of essential unsaturated fatty acids and an appropriate n-6/n-3 ratio may support both the prevention and dietary management of dermatological disorders [39,40,41,42,43,44,45].

The biological potential of hemp extends beyond its lipid profile. In vitro studies have demonstrated that hydroethanolic extracts of hemp leaves [20], aqueous extracts of inflorescences [46] and ethanolic extracts of hemp roots [47] can reduce oxidative stress-induced cellular damage and decrease the production of selected inflammatory mediators. Excessive generation of reactive oxygen species leads to damage of lipids, proteins, and genetic material, ultimately impairing normal cellular and tissue function [48,49,50]. Such processes are involved in the pathogenesis of dermatological disorders, obesity, cancer, and age-related diseases [51,52,53,54,55].

The antimicrobial activity of plant extracts has attracted considerable attention as part of the search for novel agents capable of controlling bacterial infections and limiting microbial growth in food products [56,57,58,59,60]. However, in the case of hemp, limited information is available regarding the extent to which antibacterial activity is associated with the chemical composition of different plant parts and genotype-dependent variation.

The biological properties of hemp result from the presence of numerous groups of bioactive compounds whose effects may be additive or synergistic. Their abundance depends not only on cultivar and plant part but also on climatic conditions and cultivation practices [17,19,36,61,62,63]. These factors influence both the phytochemical profile of hemp raw materials and their biological activity.

The aim of this study was to comprehensively characterize the phytochemical composition and evaluate the in vitro antibacterial activity of leaves and inflorescences from four organically grown hemp (*Cannabis sativa* L.) cultivars, providing a basis for further assessment of their potential application in dog foods. We hypothesized that hemp leaves and inflorescences would differ in their cannabinoid, terpene, and fatty acid profiles and that these differences, together with cultivar-dependent variation, would be reflected in their antibacterial activity. We further hypothesized that the compositional characteristics of hemp leaves would support their potential nutritional and functional value as a raw material for further evaluation in canine nutrition. To test these hypotheses, cannabinoid, terpene, and fatty acid profiles were determined, lipid quality indices were calculated, and the antibacterial activity of ethanolic extracts was evaluated against the reference strains *Staphylococcus aureus, Escherichia coli,* and *Pseudomonas aeruginosa*. By directly comparing two plant parts across four cultivars and integrating their chemical profiles with antibacterial activity, this study provides a broader characterization of underutilized hemp leaves and establishes a basis for further evaluation of hemp-derived materials in canine nutrition.

## 2. Materials and Methods

### 2.1. Hemp Materials

The plant material consisted of inflorescences (IF) and leaves (L) of four industrial hemp (*Cannabis sativa* L.) cultivars: Finola (F), Futura 75 (FR), Dioica (D), and Kompolti (K). All plant materials were harvested at the same developmental stage in 2024 from a certified organic plantation located in Wiekowice, Poland (54°17′59″ N, 16°21′39″ E). For each cultivar, the aerial parts of a large number of plants distributed across the plantation were manually harvested in bulk at optimal maturity. Following harvest, leaves and inflorescences were manually separated, and damaged plant parts were discarded. The leaves and inflorescences collected from each cultivar were pooled separately, resulting in one bulk sample of leaves and one bulk sample of inflorescences per cultivar. Each bulk sample was subsequently cleaned to remove soil and other foreign matter and was handled separately throughout drying, grinding, storage, and further analyses. The material was naturally air-dried in a well-ventilated room protected from direct sunlight to minimize the degradation of bioactive compounds. After drying, it was ground and stored in tightly sealed containers at room temperature under limited light exposure until analysis. Separate extraction procedures were used for cannabinoid and terpene analyses, fatty acid determination, and antibacterial activity assays, as described in the corresponding sections.

### 2.2. Determination of Cannabinoid Content

For each cultivar and plant part, the pooled plant material constituted the analytical sample. The analysis was performed in triplicate using three independently weighed subsamples from this material, each subjected separately to sample preparation and extraction. These replicates therefore represented sample-preparation/extraction technical replicates rather than independent biological replicates. Dried plant material (40 mg) was mixed with 1 mL of 96% ethanol, vortexed vigorously for 10 min, and subsequently sonicated for an additional 10 min. The samples were then centrifuged at 39,000× *g* for 5 min, and the supernatant was collected. The extraction was repeated with a fresh 1 mL portion of ethanol, and the resulting supernatants were combined and transferred into 2 mL glass vials for HPLC analysis.

Cannabinoid content determination was performed using an Agilent Technologies 1260 HPLC system (Agilent Technologies, Santa Clara, CA, USA) coupled to an Agilent 6410 triple quadrupole mass spectrometer equipped with an electrospray ionization (ESI) source. Chromatographic separation was achieved on a Supelco Ascentis Express RP-Amide column (4.6 × 100 mm) (Merck KGaA, Darmstadt, Germany). Mobile phase solvents consisted of (A) water with 0.1% formic acid and (B) acetonitrile with 0.1% formic acid. The flow rate was 0.5 mL min^−1^. The gradient program was as follows: 0 min, 75% B; 18 min, 95% B; 19 min, 95% B; 20 min, 75% B; post-run, 1 min. Total analysis time was 20 min. MS parameters were: gas temperature 300 °C, drying gas flow 12 L min^−1^, nebulizer pressure 35 psi, and capillary voltage 4000 V. 

Quantification was performed using the two most abundant MRM transitions for each analyte, as detailed in Table 1. The following cannabinoids were monitored: cannabichromene (CBC), cannabichromenic acid (CBC-A), cannabidiol (CBD), cannabidiolic acid (CBD-A), cannabigerol (CBG), cannabigerolic acid (CBG-A), cannabinol (CBN), and tetrahydrocannabinolic acid (THC-A). Analytical standards of cannabinoids were purchased from LGC Standards (Łomianki, Poland). Calibration standards were prepared over the concentration ranges of 0.5–300 µg/mL for CBC, CBC-A, CBD, CBD-A, CBG, CBG-A, and THC-A, and 0.25–300 µg/mL for CBN. Calibration curves were fitted by ordinary unweighted linear least-squares regression using LibreOffice Calc. For quantitative calculations, the regression range was restricted to calibration points encompassing the concentrations encountered in the analyzed extracts and meeting the established LOQ criterion. Accordingly, the working quantitative ranges were 1–300 µg/mL for CBD and CBC, 0.5–300 µg/mL for CBG, CBD-A, and CBG-A, 0.25–5 µg/mL for CBN, 0.5–50 µg/mL for THC-A, and 20–300 µg/mL for CBC-A. For CBD-A, CBG-A, THC-A, and CBC-A, quantification was performed using isotopically labeled internal standards, with calibration based on the analyte-to-internal-standard response ratio. CBD, CBG, CBN, and CBC were quantified using external calibration because the corresponding isotopically labeled internal standards were not available. Detailed quantitative calibration ranges, S/N values, and the estimated LOD and LOQ values for the analyzed cannabinoids are provided in Table S1 of the Supplementary Materials.

### 2.3. Determination of Volatile Terpenes Content

The same three independently prepared ethanol extracts described in Section 2.2 for cannabinoid analysis were also used for the determination of selected terpenes and terpenoids. Ethanol was selected as a broadly applicable extraction solvent because previous studies on *Cannabis sativa* have demonstrated its suitability for the recovery of both cannabinoids and terpenic compounds. Francisco et al. [64] directly compared absolute ethanol and n-hexane for the extraction of cannabis terpenes and terpenoids and indicated that ethanol is commonly used for the simultaneous recovery of cannabinoids, terpenes, and terpenoids. Addo et al. [65] specifically optimized ethanol extraction for the recovery of cannabinoids and terpenes from cannabis biomass, while Sagili et al. [66], comparing ethanol, butanol, and hexane, demonstrated that ethanol extraction provided substantial recovery of both cannabinoids and terpenes. Therefore, as the present analysis was intended for the targeted quantification of selected terpenes and terpenoids rather than exhaustive profiling of the volatile fraction, the use of the common ethanolic extract was considered suitable for this purpose.

Analyses were performed on an Agilent Technologies 5890 gas chromatograph (GC) (Agilent Technologies, Santa Clara, CA, USA) equipped with a flame ionization detector (FID) and operated with hydrogen as the carrier gas. Separation was achieved on an Agilent HP-88 capillary column (60 m × 0.25 mm i.d., 0.20 μm film thickness) (Agilent Technologies, Santa Clara, CA, USA). The suitability of the HP-88 column for the targeted analysis was verified using authentic reference standards. Under the applied chromatographic conditions, all selected terpenes and terpenoids were sufficiently resolved for identification and quantification based on retention times. The carrier gas flow rate was set to 2 mL min^−1^. The oven temperature program was as follows: initial temperature 60 °C (hold 1 min); ramp 1: 10 °C min^−1^ to 175 °C (hold 4 min); ramp 2: 10 °C min^−1^ to 210 °C (hold 2 min); post-run: 250 °C (hold 2 min). Analytical standards for terpenes were purchased from LGC Standards (Łomianki, Poland).

Calibration standards were prepared as an 11-point two-fold serial dilution covering 0.0977–100 µg/mL. For each analyte, the regression range was restricted to calibration points encompassing the concentrations encountered in the analyzed samples and meeting the established quantification criterion. The working calibration ranges were 0.0977–1.5625 µg/mL for β-myrcene and limonene, 0.78125–6.25 µg/mL for terpinene, 0.1953–6.25 µg/mL for linalool, and 0.390625–12.5 µg/mL for α-humulene. Calibration curves were fitted using unweighted linear least-squares regression. The limits of detection (LOD) and quantification (LOQ) were estimated from signal-to-noise ratios of low-concentration calibration standards, using S/N = 3 and S/N ≥ 10 as the respective criteria. LOQ was conservatively assigned as the lowest experimentally measured calibration standard meeting the S/N ≥ 10 criterion. Detailed quantitative calibration ranges, S/N values, and the estimated LOD and LOQ values for individual terpenes and terpenoids are provided in Table S2 of the Supplementary Materials.

### 2.4. Determination of Fatty Acid Methyl Esters (FAMEs)

All reagents and FAME standards were purchased from Merck Life Science Sp. z o.o. (affiliate of Merck KGaA, Darmstadt, Germany). Dried plant material (0.5 g) was extracted with chloroform. Each sample was mixed with 3 mL of chloroform, sonicated for 30 min, and subsequently agitated on a rotary shaker for 12 h. The samples were then centrifuged (3000× *g*, 10 min), and the supernatant was collected. The extraction was repeated with an additional 3 mL of fresh chloroform, and the two supernatants were combined.

A 2 mL aliquot of the combined chloroform extract was transferred into Pyrex screw-cap tubes and mixed with 2 mL of 20% (*v*/*v*) sulfuric acid in methanol. Samples were heated at 100 °C for 1 h in a heating block to achieve transmethylation. After cooling to room temperature, 1 mL of water was added, and the mixture was vigorously vortexed. Following phase separation, the lower chloroform layer was collected, passed through a short cotton plug to remove residual moisture and particulates, and transferred into 2 mL glass GC vials. Determination of the fatty acid methyl esters (FAMEs) was analyzed using an Agilent Technologies 5890 gas chromatograph equipped with a flame ionization detector (FID) and operating with hydrogen as the carrier gas. Chromatographic separation was performed on an Agilent HP-88 capillary column (60 m × 0.25 mm i.d., 0.20 μm film thickness). The carrier gas flow rate was 2 mL min^−1^. The oven temperature program was as follows: initial temperature 120 °C (hold 1 min); ramp 1: 10 °C min^−1^ to 175 °C (hold 8 min); ramp 2: 5 °C min^−1^ to 210 °C (hold 3 min); post-run: 250 °C (hold 2 min). The injection volume was 1 μL.

FAMEs were identified by comparison of their retention times with authentic reference standards (Supelco 37 Component FAME Mix). Fatty-acid composition was expressed as the relative percentage of each identified fatty acid in the total identified fatty-acid fraction. Internal standards were not used because the fatty-acid analysis was intended to determine the relative composition of the FAME fraction rather than absolute analyte concentrations. Individual FAME peak areas were converted to amounts using analyte-specific external calibration curves. The mass of each quantified fatty acid was then expressed as a percentage of the total mass of all quantified fatty acids. Detector-response linearity was assessed using the same analytical standard. An eight-point two-fold serial dilution series (L0–L7), corresponding to a 128-fold concentration range, was used for unweighted linear least-squares regression. An additional dilution level (L8) was prepared but was excluded from the regression because the concentrations of several analytes were below the useful quantitative response range. The coefficients of determination (R^2^) for the individual FAMEs ranged from 0.9949 to 0.9990. Signal-to-noise ratios were determined at dilution level L6 (64-fold dilution), and the limits of detection (LOD) and quantification (LOQ) were estimated using S/N ratios of 3 and 10, respectively. Analyte-specific calibration ranges, R^2^ values, and estimated LOD and LOQ values for the fatty acids analyzed in the present study are provided in Table S3 of the Supplementary Materials. The variability among sample-preparation/extraction technical replicates, expressed as relative standard deviation (RSD) for the major fatty acids, is presented in Table S4 of the Supplementary Materials.

### 2.5. Lipid Quality Indices

Based on the fatty acid profile, the following lipid quality indices were calculated: desirable fatty acids (DFA), undesirable fatty acids (OFA), the index of atherogenicity (AI), the index of thrombogenicity (TI), the hypocholesterolemic-to-hypercholesterolemic fatty acid ratio (h/H), and the health-promoting index (HPI) (Equations (1)–(6)). The AI and TI were calculated according to the equations proposed by Ulbricht and Southgate [67], the h/H ratio according to Santos-Silva et al. [68], and the HPI according to Chen et al. [69]. The definition and calculation of OFA were adopted from Chen and Liu [70].

(1) Desirable Fatty Acids (DFA)=C18:1 n−9+ΣPUFA

(2) Hypercholesterolemic Fatty Acids (OFA)=C12:0+C14:0+C16:0

(3) $$\mathrm{Index}\;\mathrm{of}\;\mathrm{Atherogenicity}\;(AI)=\frac{C12:0+4\times C14:0+C16:0}{\Sigma UFA+C18:0}$$

(4) $$\mathrm{Index}\;\mathrm{of}\;\mathrm{Thrombogenicity}\;(\mathrm{TI})=\frac{C14:0+C16:0+C18:0}{\left(0.5\times C18:1)+(0.5\times other\;MUFA)+(0.5\times \Sigma n-6\;PUFA)+(3\times \Sigma n-3\;PUFA)+(\frac{\Sigma n-3\;PUFA}{\Sigma n-6\;PUFA}\right)}$$

(5) $$Hypocholesterolemic/Hypercholesterolemic\;Fatty\;Acid\;Ratio\;(h/H)=\frac{C18:1+\Sigma PUFA}{C12:0+C14:0+C16:0}$$

(6) $$\mathrm{Health}\text{-}\mathrm{Promoting}\;\mathrm{Index}\;(\mathrm{HPI})=\frac{\Sigma \mathrm{UFA}}{C12:0+(4\;\times \;C14:0)+C16:0}$$

### 2.6. Evaluation of the Antibacterial Activity of Hemp Extracts

#### 2.6.1. Preparation of Hemp Extracts

For the evaluation of antibacterial activity, extracts were prepared using 75% ethanol as the extraction solvent. The dried plant material was macerated for 7 days at room temperature in the absence of light using a shaking water bath (FWS30, CHEMLAND, Stargard, Poland) operated at 270 rpm. Following extraction, the mixtures were filtered through filter paper into pre-dried flasks, and the solvent was removed by evaporation. The extracts were subsequently dried in a laboratory oven for 12 h. The extracts were then transferred into sterile Falcon tubes and suspended in 99% dimethyl sulfoxide (DMSO; analytical grade, 99.9%; ChemWorld, Emspit, Szczecin, Poland) and adjusted to obtain a final working concentration of 25% and transferred into sterile Falcon tubes. Before preparing serial dilutions, each suspension was thoroughly mixed.

#### 2.6.2. Determination of Minimum Inhibitory Concentration (MIC) and Minimum Bactericidal Concentration (MBC)

The minimum inhibitory concentration (MIC) of the hemp extracts was determined using the broth microdilution method in Mueller–Hinton broth (MHB) from Graso Biotech Sp. z o.o. (Starogard Gdański, Poland). Two-fold serial dilutions of the extracts were prepared in the concentration range of 2.5–0.005% (*v*/*v*) in 96-well microtiter plates (NEST Biotechnology, Wuxi, China). Each well was inoculated with a bacterial suspension to obtain a final concentration of 1 × 10^6^ CFU/mL. Three reference strains were used: *Staphylococcus aureus* ATCC 6538, *Escherichia coli* ATCC 11303, and *Pseudomonas aeruginosa* PCM 2710. All strains were obtained from the Culture Collection of the Department of Microbiology and Biotechnology, West Pomeranian University of Technology in Szczecin, Poland. Positive (bacterial growth without extract) and negative (sterility control of MHB) controls were included in each assay. The plates were incubated at 37 °C for 18 h, after which the MIC was defined as the lowest extract concentration that completely inhibited visible bacterial growth, as indicated by the absence of turbidity. Each concentration was tested in triplicate. To evaluate the intrinsic antibacterial activity of the solvent, pure DMSO was additionally tested against each bacterial strain over a concentration range of 0–100% (*v*/*v*). Because the intense color of the extracts interfered with the visual assessment of bacterial growth, the contents of each well were spread onto Columbia Agar supplemented with 5% sheep blood (Argenta Sp. z o.o., Poznań, Poland). Following incubation at 37 °C for 18 h, bacterial colony growth was evaluated. The minimum bactericidal concentration (MBC) was defined as the lowest extract concentration at which no bacterial colonies were observed on the agar plates.

#### 2.6.3. Time–Kill Assay

The bactericidal activity of the hemp extracts over time was evaluated using a time–kill assay performed at the MIC and/or MBC values determined for each reference bacterial strain (*S. aureus*, *E. coli*, *P. aeruginosa*). Each tube (n = 8 per bacterium) contained a final volume of 1 mL of Mueller–Hinton broth supplemented with the appropriate concentration of the hemp extract and inoculated with the test organism to obtain a final bacterial concentration of 1 × 10^6^ CFU/mL. For each bacterial strain, two control samples consisting of MHB alone and MHB supplemented with 5% DMSO (without extract but with the same bacterial inoculum) were included. The tubes were immediately incubated at 37 °C, and 100 μL aliquots were collected from each tube after a certain period of time: 15 min, 30 min, 1, 2, 4, 6, 8, 12, and 24 h. Each sample was subjected to ten-fold serial dilutions in sterile physiological saline (Ecotainer^®^, B. Braun Medical AG, Sempach, Switzerland) and immediately, 100 μL of each dilution was spread onto Columbia Agar supplemented with 5% sheep blood (Argenta Sp. z o.o., Poznań, Poland). On a blood agar medium, the specific morphology of the colonies and hemolysis are clearly visible, and this allows for the elimination of possible contamination by another bacterium, which could result in a false-positive result. The plates were incubated at 37 °C for 24 h, after which the number of viable bacteria (expressed as CFU/mL) was determined by manual colony counting, taking into account the dilution ratio and the sample volume collected.

### 2.7. Statistical Analysis

A two-factorial analysis of variance (ANOVA) and principal component analysis (PCA) were carried out using the STATISTICA v13.30 software (TIBCO Software Inc., Palo Alto, CA, USA). Tukey’s honestly significant difference (HSD) at *p* = 0.05 was used to find the differences between means. Values for cultivars represent means across leaves and inflorescences, whereas values for plant parts represent means across all cultivars.

## 3. Results

### 3.1. Cannabinoid Content

The phytochemical composition of plant materials may vary depending on numerous factors, including cultivar, developmental stage, plant part, environmental and agronomic conditions, as well as harvesting, transportation, and storage conditions [17,61,62].

The total content of individual cannabinoid groups differed significantly among the hemp cultivars studied (Table 2). The Kompolti cultivar contained a significantly lower total cannabinoid content than the remaining cultivars, among which no significant differences were observed. The highest total CBC content was found in the Dioica cultivar, whereas the lowest values were recorded in Finola and Kompolti. Futura 75 and Dioica exhibited the highest total CBD content, while Kompolti contained significantly lower amounts of this cannabinoid. An opposite trend was observed for total CBG, with the highest concentration detected in Kompolti and the lowest in Futura 75 and Dioica. Plant part also had a significant effect on the total content of all cannabinoid groups. Regardless of cultivar, inflorescences contained significantly higher amounts of total CBC, CBD and CBG than leaves, resulting in a significantly greater total cannabinoid content.

The concentrations of individual cannabinoids are presented in Table 3. Acidic cannabinoids predominated in all plant materials, with CBD-A and CBG-A being the most abundant compounds. The highest CBD-A concentration was detected in Futura 75 and Dioica, whereas the lowest was found in Kompolti. A similar pattern was observed for CBD. In contrast, Kompolti contained the highest concentrations of CBC, CBG, and CBG-A, whereas Futura 75 was characterized by the highest THC-A and CBN contents. The concentrations of all analyzed cannabinoids were also significantly affected by plant part. When averaged across cultivars, inflorescences contained significantly higher amounts of each cannabinoid than leaves. The greatest differences were observed for CBD-A and CBG-A, whose concentrations in inflorescences were approximately 2.8- and 3.0-fold higher, respectively, than in leaves.

The cultivar-specific distribution of individual cannabinoids between leaves and inflorescences is presented in Supplementary Figures S2–S9. These figures allow direct comparisons of plant organs within each *Cannabis sativa* L. cultivar.

### 3.2. Volatile Terpenes Content

Due to the volatile nature of terpenes, their concentrations in plant materials may be affected by drying and storage conditions. Among all terpenes analyzed, α-humulene was the predominant compound, with concentrations ranging from 173.154 to 303.483 μg/g DM, depending on the cultivar (Table 4). The highest α-humulene concentration was recorded in the Finola cultivar, whereas the lowest was observed in Futura 75. The Dioica cultivar was characterized by significantly higher concentrations of β-myrcene, limonene, and linalool than the remaining cultivars. Regardless of cultivar, inflorescences contained significantly higher concentrations of α-humulene, β-myrcene, limonene, and linalool than leaves, whereas terpinen was significantly more abundant in leaves. The greatest differences between plant parts were observed for linalool and α-humulene, whose concentrations in inflorescences were approximately 3.6- and 2.2-fold higher, respectively, than in leaves.

The cultivar-specific distribution of individual terpenes between leaves and inflorescences is presented in Supplementary Figures S10–S14. These figures enable direct comparisons of leaves and inflorescences within each *Cannabis sativa* L. cultivar.

**Figure 1:**
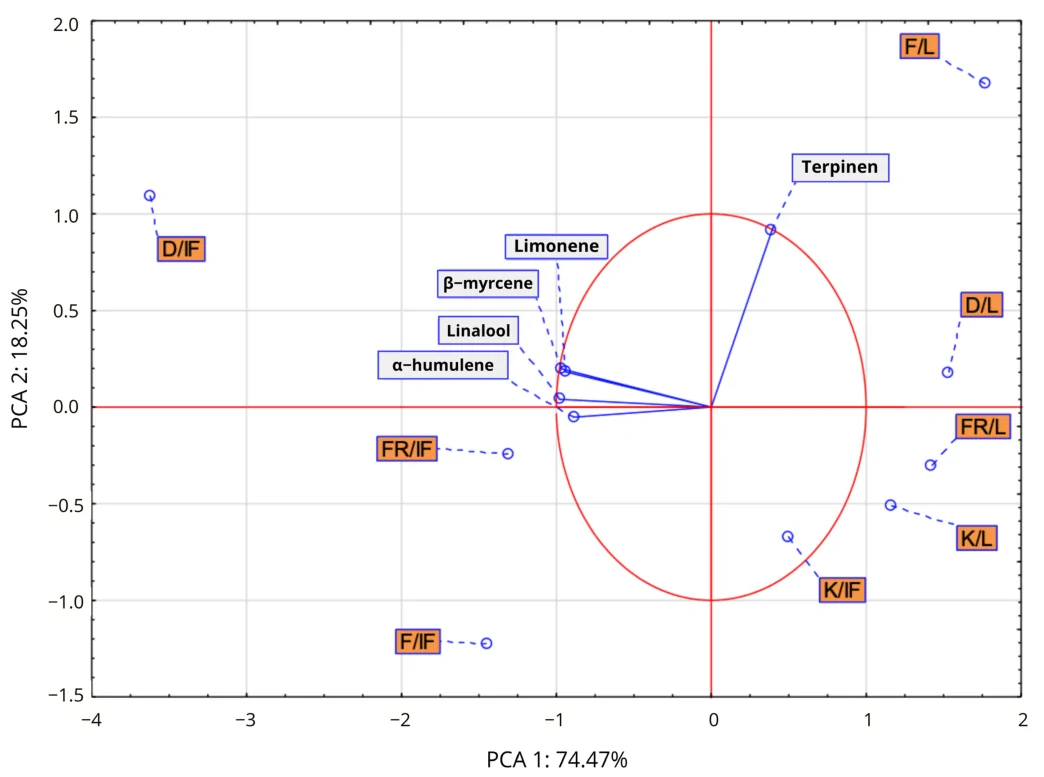
Biplot based on the first two principal component axes for the terpene composition of raw material (leaves and inflorescences) from four cultivars of hemp; D—cv. Dioica, F—cv. Finola, FR—cv. Futura 75, K—cv. Kompolti; IF—inflorescences; L—leaves.

Principal component analysis (PCA) showed that the first two principal components explained 92.72% of the total variance (Figure 1). The PCA clearly demonstrated differences in terpene profiles among the hemp cultivars. The loading plot revealed that only the terpinen vector was located in the first quadrant, indicating a markedly higher abundance of this compound in the leaves of the Finola and Dioica cultivars. The remaining terpene vectors were nearly parallel to one another and approximately perpendicular to the terpinen vector, indicating that most terpenes, with the exception of terpinen, were associated with the inflorescences of the Dioica, Futura 75, and Finola cultivars. Furthermore, the PCA indicated no significant correlations between terpinen and the other terpenes analyzed.

### 3.3. Fatty Acid Composition and Lipid Quality Indices

The fatty acid composition of the analyzed hemp cultivars and plant parts is presented in Table 5, whereas the proportions of the major fatty acid groups and the n-6/n-3 ratio are summarized in Table 6. The calculated lipid quality indices are presented in Table 7. Among saturated fatty acids (SFA), palmitic acid (C16:0) was the predominant fatty acid regardless of cultivar or plant part. The highest C16:0 concentrations were recorded in the Futura 75 and Dioica cultivars, whereas the lowest concentration was observed in Finola. Futura 75 and Dioica were also characterized by significantly higher concentrations of stearic acid (C18:0). In contrast, the concentration of lignoceric acid (C24:0) did not differ significantly among cultivars. Finola contained the lowest concentration of arachidic acid (C20:0), which was consistent with its overall lower SFA content. Comparison of plant parts showed that inflorescences contained significantly higher concentrations of C16:0, C18:0, and C20:0, whereas leaves were characterized by significantly higher proportions of C12:0, C14:0, and C24:0.

Among unsaturated fatty acids, α-linolenic acid (C18:3 n-3) was the predominant fatty acid, accounting for 33.45–44.53% of the total fatty acid pool, with the lowest and highest values recorded in Futura 75 and Finola, respectively. Finola and Dioica also contained the highest concentrations of linoleic acid (C18:2 n-6). Regarding monounsaturated fatty acids, oleic acid (C18:1 n-9) was most abundant in Futura 75, whereas palmitoleic acid (C16:1 n-7) reached its highest concentration in Dioica. Significant differences were also observed between plant parts. Inflorescences contained higher concentrations of oleic and linoleic acids, whereas leaves contained higher concentrations of palmitoleic and α-linolenic acids. Overall, leaves were characterized by significantly higher proportions of α-linolenic, lauric (C12:0), myristic (C14:0), palmitoleic, and lignoceric acids, whereas inflorescences contained significantly higher proportions of linoleic, oleic, and stearic acids.

**Figure 2:**
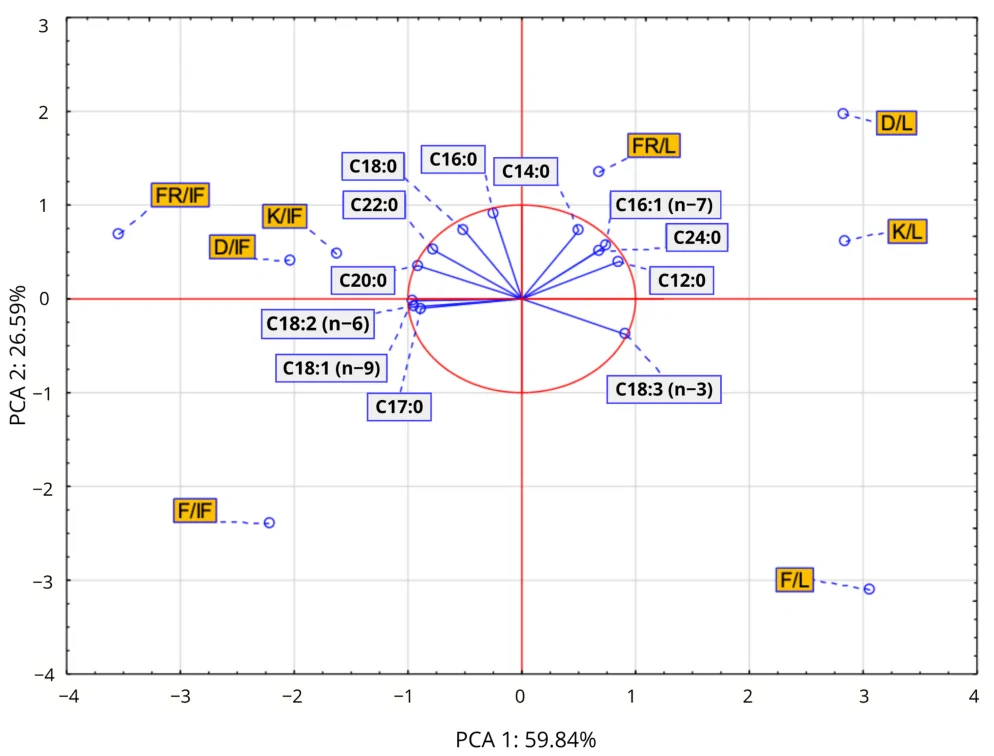
Biplot based on the first two principal component axes for the fatty acid composition of raw material (leaves and inflorescences) from four hemp cultivars; D—cv. Dioica, F—cv. Finola, FR—cv. Futura 75, K—cv. Kompolti; IF—inflorescences; L—leaves.

Principal component analysis (PCA) showed that the first two principal components explained 86.43% of the total variance (Figure 2). The analysis confirmed clear differences in fatty acid composition among the analyzed hemp raw materials. As shown in the PCA plot, the leaves of all cultivars were located in the first and fourth quadrants. In particular, the leaves of the Finola cultivar were associated with a higher content of C18:3 n-3, whereas the leaves of the remaining cultivars were characterized by higher contents of C12:0, C14:0, C24:0, and C16:1. These results indicate that Finola leaves had a distinct fatty acid profile compared with the leaves of the other cultivars. The inflorescences of the analyzed cultivars were located in the second and third quadrants. Among them, the Finola inflorescences were separated from the remaining cultivars by their position in the third quadrant, whereas the other inflorescences clustered in the second quadrant and were associated with slightly higher contents of C18:0, C20:0, and C22:0.

Polyunsaturated fatty acids (PUFA) were the predominant fatty acid group in all hemp raw materials, accounting for 49.593–59.554% of the total fatty acid pool. Finola exhibited the highest PUFA and n-3 fatty acid contents, whereas Futura 75 contained the highest proportions of saturated (SFA) and monounsaturated fatty acids (MUFA). The highest n-6/n-3 ratio was observed in Futura 75 and Dioica, while Kompolti was characterized by the lowest n-6 fatty acid content. Plant part also significantly affected fatty acid composition. Leaves contained significantly higher proportions of PUFA and n-3 fatty acids, whereas inflorescences contained significantly higher proportions of SFA, MUFA, and n-6 fatty acids. Consequently, the n-6/n-3 ratio in leaves was more than two-fold lower than that observed in inflorescences (0.241 vs. 0.628).

To comprehensively evaluate lipid quality, several nutritional and health-related indices were calculated based on the fatty acid composition (Equations (1)–(6)). These indices reflected the potential atherogenic and thrombogenic properties of the lipid fraction, as well as the proportion of nutritionally desirable fatty acids. Finola exhibited the lowest AI and TI values, whereas the highest values were observed in Futura 75. A similar trend was found for OFA. In contrast, Finola showed the highest h/H, HPI, and DFA values, whereas the lowest values were recorded in Futura 75 and Dioica. With respect to plant part, leaves exhibited lower AI and TI values and higher h/H, HPI, and DFA values than inflorescences. No significant differences in OFA values were observed between leaves and inflorescences.

**Figure 3:**
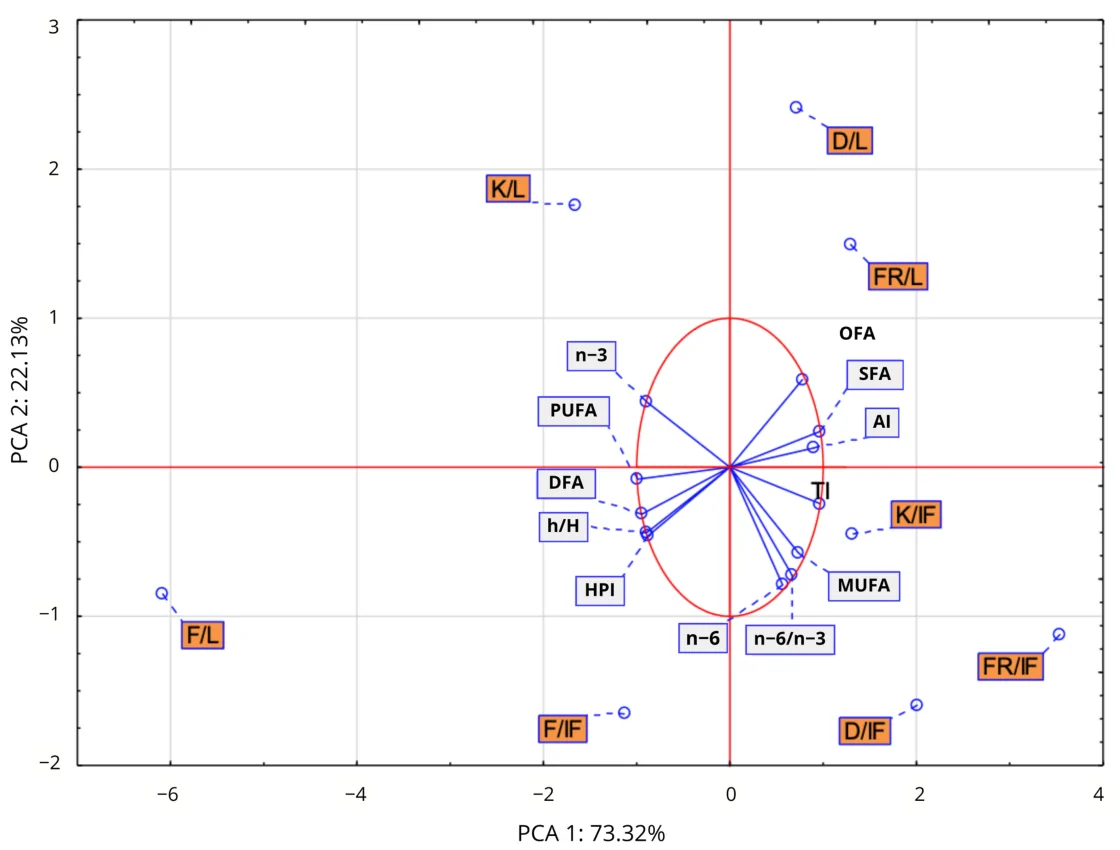
Biplot based on the first two principal component axes for lipid indices and fatty acid groups of leaves and inflorescences from four hemp (*Cannabis sativa* L.) cultivars; D—cv. Dioica, F—cv. Finola, FR—cv. Futura 75, K—cv. Kompolti, IF—inflorescences, L—leaves, DFA—desirable fatty acids, OFA—hypercholesterolemic fatty acids, AI—index of atherogenicity, TI—index of thrombogenicity, h/H—hypocholesterolemic/hypercholesterolemic fatty acid ratio, SFA—saturated fatty acids, MUFA—monounsaturated fatty acids, PUFA—polyunsaturated fatty acids.

Principal component analysis (PCA) showed that the first two principal components explained 95.45% of the total variance (Figure 3). The analysis confirmed differences among the analyzed hemp raw materials with respect to lipid quality indices. Plant part had a greater influence on sample distribution than cultivar. The inflorescences of three cultivars were located in the fourth quadrant, whereas the Finola inflorescences were positioned in the third quadrant, close to the remaining inflorescences. This indicates that Finola inflorescences were associated with slightly higher HPI, h/H, and DFA values and lower n-6, n-6/n-3, and MUFA values than the other inflorescences. In contrast, the leaves of the analyzed cultivars were distributed across three quadrants, indicating greater variation in lipid quality indices among cultivars. Only the leaves of Dioica and Futura 75 were located in the first quadrant, indicating higher OFA, SFA, and AI values than those of the remaining cultivars.

### 3.4. Antibacterial Activity

#### 3.4.1. Minimum Inhibitory Concentration (MIC) and Minimum Bactericidal Concentration (MBC)

The antibacterial activity of the hemp extracts was evaluated against the reference strains *Staphylococcus aureus*, *Escherichia coli*, and *Pseudomonas aeruginosa* (Table 8). Among the tested bacteria, *S. aureus* was the most susceptible to the hemp extracts, with all extracts exhibiting identical MIC and MBC values of 0.005% (*v*/*v*). Similarly, no differences among the extracts were observed for *E. coli*, for which both MIC and MBC were 0.625% (*v*/*v*). *P. aeruginosa* was the least susceptible strain. The strongest antibacterial activity was observed for extracts prepared from the inflorescences of the Finola and Kompolti cultivars, with MIC and MBC values of 1.25% (*v*/*v*). The remaining extracts inhibited bacterial growth and exhibited bactericidal activity against *P. aeruginosa* at 2.5% (*v*/*v*). For leaf extracts of the Finola and Kompolti cultivars, the MIC values were lower than the corresponding MBC values, amounting to 1.25% and 2.5% (*v*/*v*), respectively.

DMSO at a concentration of 5% (*v*/*v*) had no effect on the growth of the tested bacterial strains. The MIC and MBC values of the solvent alone were 10% (*v*/*v*) for *S. aureus* and 10–20% (*v*/*v*) for the Gram-negative bacteria, indicating that the observed antibacterial activity resulted from the hemp extracts rather than the solvent.

#### 3.4.2. *Time–Kill Kinetics*

**Figure 4:**
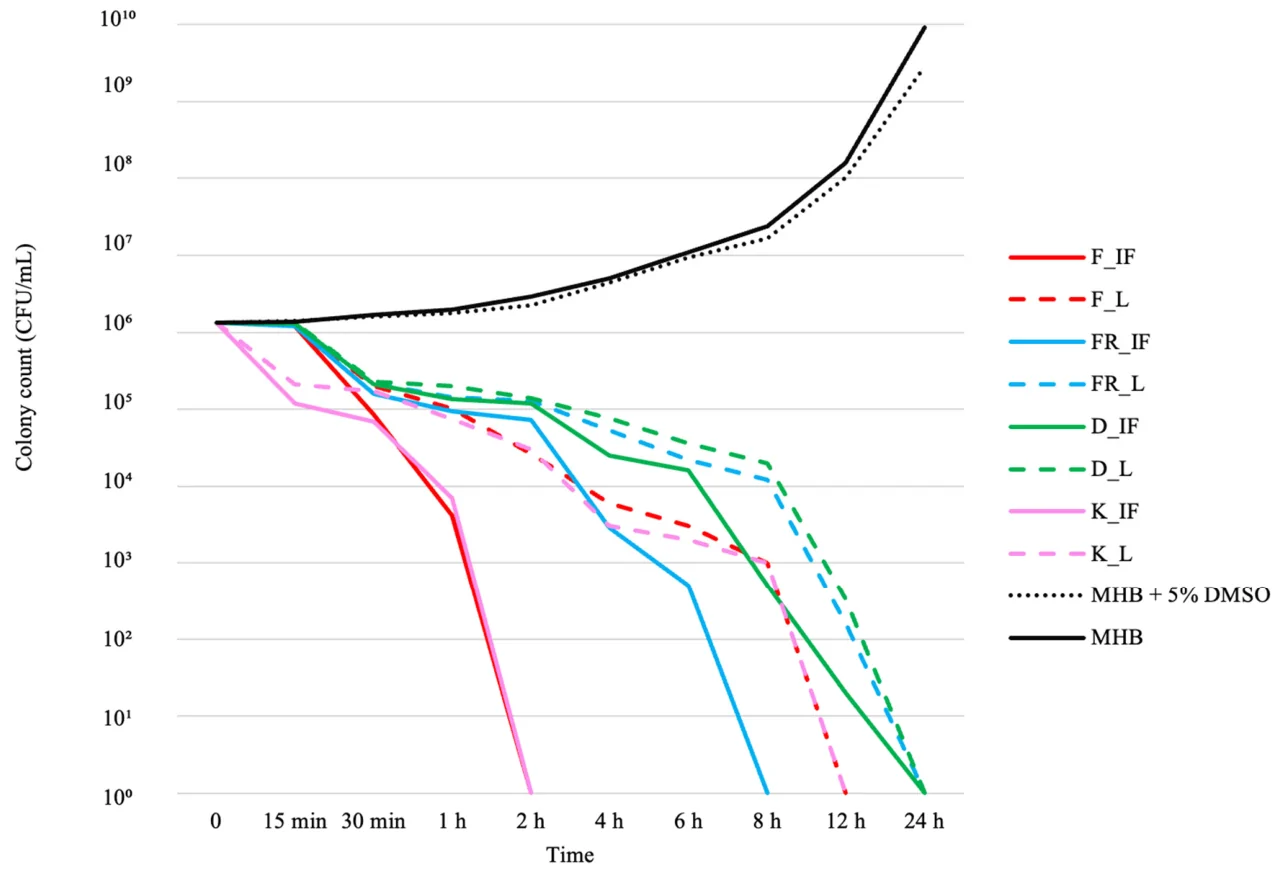
Time–kill curves of ethanolic extracts against *Staphylococcus aureus* ATCC 6538 at 0.005% *v*/*v* (1 × MIC). Complete bacterial elimination was observed after 2 h for F_IF and K_IF, after 8 h for FR_IF, after 12 h for F_L and K_L, and after 24 h for the remaining extracts.

**Figure 5:**
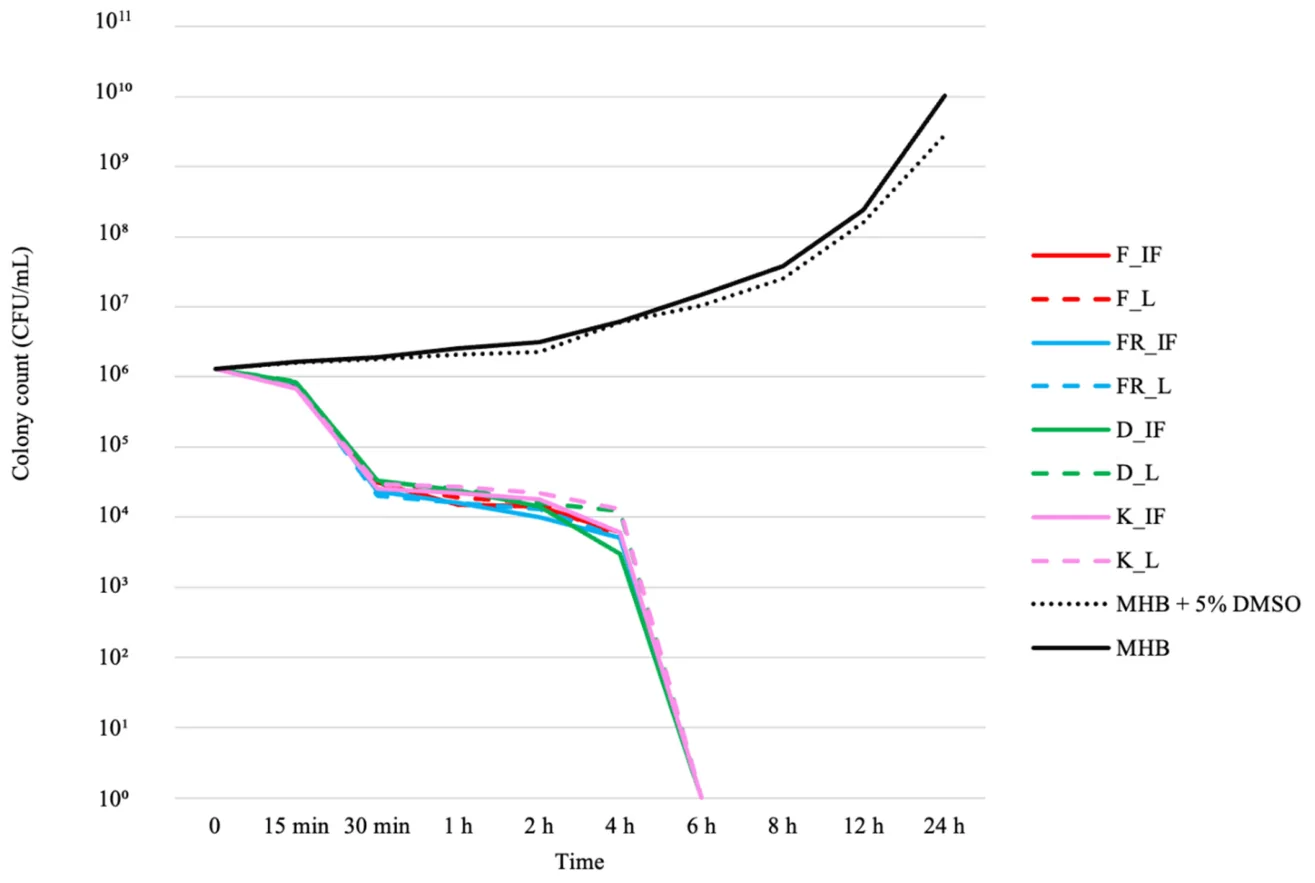
Time–kill curves of ethanolic extracts against *Escherichia coli* ATCC 11303 at 0.625% *v*/*v* (1 × MIC). Complete bacterial elimination occurred after 6 h for all extracts.

**Figure 6:**
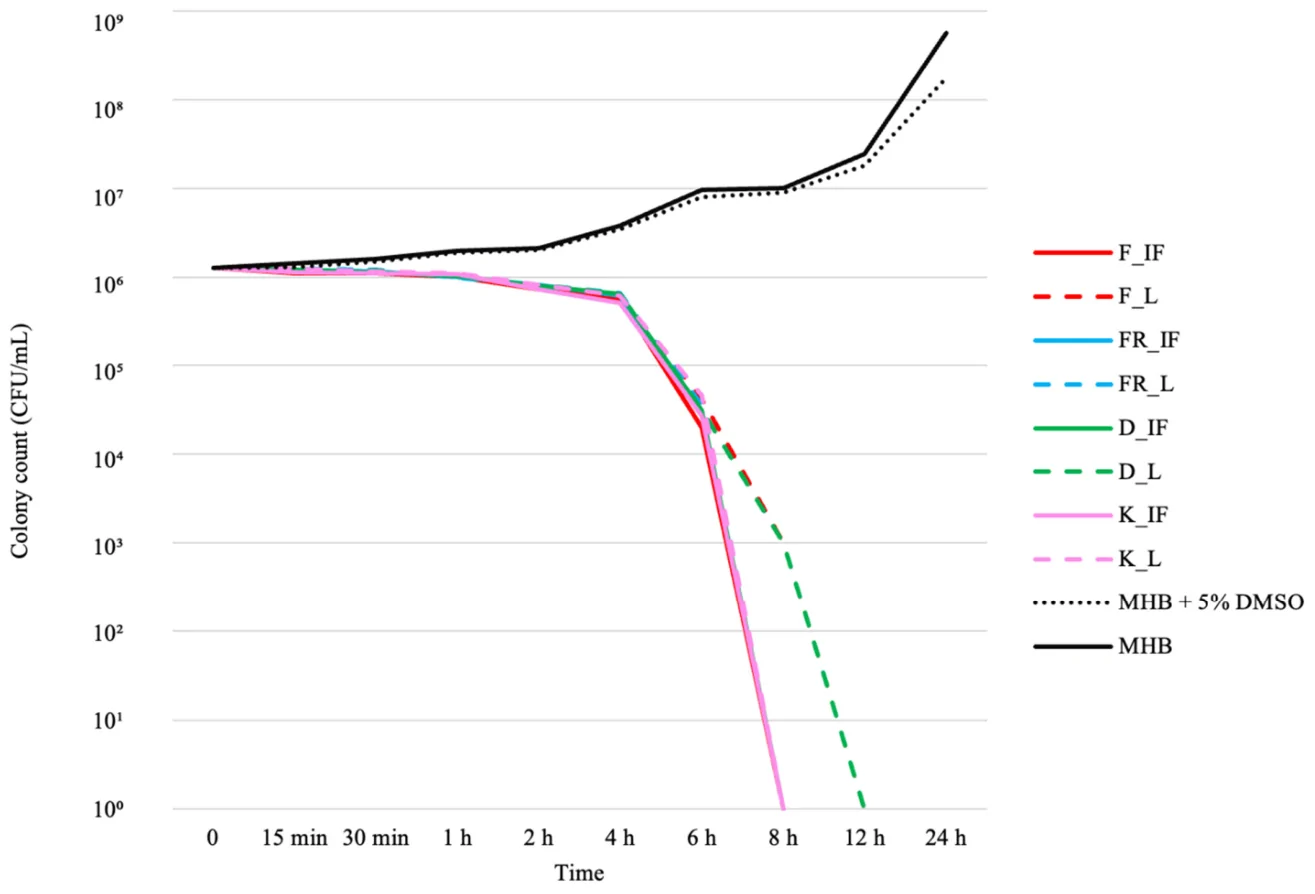
Time–kill curves of ethanolic extracts against *Pseudomonas aeruginosa* PCM 2710. F_IF and K_IF extracts were tested at 1.25% *v*/*v* mL (1 × MIC), whereas the remaining extracts were tested at 2.5% (1 × MIC). Complete bacterial elimination occurred after 8 h for F_IF, FR_IF, FR_L, D_IF, K_IF, and K_L, and after 12 h for F_L and D_L.

The results of the time–kill assay are presented in Figure 4, Figure 5 and Figure 6. All extracts were tested at concentrations corresponding to their MIC and MBC values. The highest antibacterial activity against *S. aureus* was observed for the inflorescence extracts of the Finola and Kompolti cultivars (F_IF and K_IF), which completely eliminated bacterial growth after only 2 h of incubation (Figure 4). The corresponding leaf extracts required 12 h to achieve complete bacterial elimination. For the Futura 75 cultivar, the inflorescence extract exhibited bactericidal activity after 8 h, whereas the leaf extract required 24 h. The slowest bactericidal activity was observed for the Dioica extracts (D_IF and D_L) and the Futura 75 leaf extract (FR_L), for which complete bacterial elimination was achieved only after 24 h of incubation. The time–kill analysis performed against *Escherichia coli* (Figure 5) demonstrated a more uniform antibacterial effect than that observed for *S. aureus*. Regardless of cultivar or plant part, all extracts completely eliminated bacterial growth after 6 h of incubation at a concentration of 0.625% (*v*/*v*). For *Pseudomonas aeruginosa*, differences were observed in both the effective concentrations and the rate of bactericidal activity (Figure 6). The F_IF and K_IF extracts, tested at 1.25% (*v*/*v*) (1 × MIC), completely eliminated bacterial growth after 8 h of incubation. At 2.5% (*v*/*v*) (1 × MIC), complete bacterial elimination after 8 h was also observed for the FR_IF, FR_L, D_IF, and K_L extracts. The slowest antibacterial activity was exhibited by the F_L and D_L extracts, which completely eliminated *P. aeruginosa* only after 12 h of incubation.

## 4. Discussion

The biological activity of hemp results from the combined presence of numerous primary nutrients and secondary metabolites and therefore cannot be attributed solely to individual cannabinoids. Increasing evidence suggests that the biological properties of hemp are determined by interactions among cannabinoids, terpenes, phenolic compounds, and other constituents present in the plant material. This phenomenon, referred to as the *entourage effect*, proposes that the combined action of multiple compounds produces biological effects that differ from those of isolated constituents [71]. Although the molecular mechanisms underlying this effect have not yet been fully elucidated, its importance is increasingly recognized in industrial hemp, where biological activity appears to depend on the overall phytochemical profile rather than exclusively on the concentration of the major cannabinoids [72].

The results of the present study confirmed that acidic cannabinoids, particularly CBD-A and CBG-A, predominated in all analyzed hemp cultivars. Moreover, the total cannabinoid content was nearly threefold higher in inflorescences than in leaves. Similar observations were reported by Jin et al. [16] and Stack et al. [62], who demonstrated that both genotype and cultivation conditions significantly influence the cannabinoid profile. In veterinary medicine, cannabinoid-containing preparations are increasingly being investigated as supportive agents in the management of chronic pain, epilepsy, and inflammatory disorders in dogs. In a 90-day clinical trial involving dogs with osteoarthritis, dietary supplementation with a hemp extract improved pain control in 30 of 32 animals, while most dogs receiving concomitant gabapentin treatment were able to reduce the dose or discontinue the medication entirely [73,74]. Beneficial effects have also been reported in dogs with idiopathic epilepsy, in which administration of CBD or a combination of CBD and CBD-A reduced seizure frequency, with more than a 50% reduction observed in some animals [73,75]. Furthermore, CBD reduced the production of pro-inflammatory cytokines (IL-1β, IL-6, and TNF-α) in canine blood cells, whereas treatment of dogs with drug-resistant epilepsy was associated with decreased circulating TNF-α concentrations [76]. Despite these promising findings, the practical application of cannabinoids in canine nutrition requires careful consideration. CBD-rich formulations are generally well tolerated by dogs; however, long-term administration should be accompanied by monitoring of biochemical parameters, particularly alkaline phosphatase (ALP) activity [73,77,78]. Reported alterations in these parameters appear to depend largely on the administered dose, duration of treatment, and formulation of the cannabinoid preparation. It should also be emphasized that most published studies have evaluated highly concentrated cannabinoid formulations administered at therapeutic doses, which greatly exceed the amounts that would be supplied through conventional diets [73,74,75,76,77,78]. Consequently, the safety of hemp leaves and inflorescences as feed ingredients requires independent evaluation under practical dietary conditions, considering the actual intake and bioavailability of cannabinoids. Pharmacokinetic studies in dogs have demonstrated that CBD-A is efficiently absorbed following oral administration and that its bioavailability may be enhanced by appropriate formulation strategies [78]. The predominance of acidic cannabinoids observed in the present study was most likely a consequence of the mild drying conditions, which limited the decarboxylation of naturally occurring cannabinoid acids. Therefore, processing conditions may play an important role in preserving the cannabinoid profile and, consequently, the functional properties of hemp materials intended for incorporation into canine diets.

The high α-humulene content observed in the analyzed hemp raw materials, together with the presence of β-myrcene, limonene, and linalool, further enhances the biological potential of the obtained extracts. These terpenes exhibit both anti-inflammatory and antioxidant properties. α-humulene inhibits activation of the NF-κB signaling pathway, which plays a key role in regulating the inflammatory response [79,80,81]. Chronic inflammatory disorders, such as canine atopic dermatitis and osteoarthritis, are among the conditions in which these terpenes may have practical therapeutic potential.

One of the most pronounced differences between the analyzed plant parts was their fatty acid composition. Leaves contained significantly higher levels of α-linolenic acid (ALA) and exhibited a more favorable n-6/n-3 fatty acid ratio than inflorescences. This fatty acid profile supports the potential use of hemp leaves as functional ingredients in companion-animal diets. Appropriate proportions of essential fatty acids (EFA) regulate inflammatory processes, influence the lipid composition of cell membranes, and contribute to maintaining healthy skin and coat condition [82,83,84,85]. Accordingly, dietary management based on an appropriate unsaturated fatty acid profile represents one of the fundamental components of therapy for dogs with atopic dermatitis. Analyses of commercial dog foods have demonstrated that both fatty acid composition and the n-6/n-3 ratio may vary considerably among products, regardless of their market price [82,83,86,87]. The biological significance of the n-6/n-3 ratio is related to the fact that both fatty acid families share the same metabolic enzymes, particularly Δ6-desaturase. Competition between linoleic acid (LA, n-6) and α-linolenic acid (ALA, n-3) affects the synthesis of long-chain polyunsaturated fatty acids and their downstream inflammatory mediators [88]. Consequently, a high dietary proportion of n-6 fatty acids may limit the conversion of ALA into its long-chain derivatives. Therefore, the biological value of dietary lipids depends not only on the amount of n-3 fatty acids but also on their proportion relative to n-6 fatty acids [88,89]. Vastolo et al. [90] demonstrated that dietary supplementation with hempseed cake, which contains considerably lower concentrations of bioactive compounds than hemp leaves or inflorescences, increased the dietary content of linoleic and α-linolenic acids while reducing the n-6/n-3 ratio. These changes were accompanied by reductions in serum cholesterol, ALT, AST, and creatinine concentrations. Given the lower abundance of bioactive compounds in hempseed cake compared with leaves and inflorescences, the present findings support further investigation of aerial hemp tissues as potential functional feed ingredients for companion animals. The more favorable fatty acid profile of hemp leaves was also reflected in their lipid quality indices. Lower AI and TI values together with higher h/H and HPI values indicate a more favorable lipid profile in leaves than in inflorescences. Although these indices were originally developed to evaluate the nutritional quality of lipids for human consumption, they are increasingly being applied to characterize feed raw materials. In canine nutrition, however, these indices should be interpreted primarily in the context of the underlying fatty acid profile, particularly PUFA proportions and the n-6/n-3 ratio, for which evidence regarding effects on lipid metabolism and inflammatory processes is stronger [91]. Therefore, the AI, TI, h/H, and HPI values obtained in the present study should be regarded as indicators of the more favorable lipid profile of hemp leaves rather than as independent measures of their nutritional value.

Staphylococcal infections are among the most frequently diagnosed bacterial diseases in dogs. Although the present study employed the reference strain *Staphylococcus aureus*, the predominant bacterial species responsible for skin, ear, and wound infections in dogs is *Staphylococcus pseudintermedius* [92]. The increasing prevalence of methicillin-resistant *S. pseudintermedius* (MRSP), characterized by resistance to multiple classes of antibiotics, highlights the need to identify novel antibacterial agents [92,93]. Therefore, the present findings support further investigations into the antibacterial activity of hemp extracts against *S. pseudintermedius*, including MRSP strains.

The greater susceptibility of Gram-positive bacteria compared with Gram-negative bacteria observed in the present study is consistent with previous reports. Ali et al. [94] demonstrated that both hemp seed oil and whole-plant *Cannabis sativa* extracts effectively inhibited the growth of *Staphylococcus aureus*, whereas substantially weaker activity against *Escherichia coli* and *Pseudomonas aeruginosa* was observed, depending on the type of extract used. Similar findings were reported by Muscarà et al. [95] who demonstrated potent antibacterial activity of hemp inflorescence extracts against both reference and methicillin-resistant *S. aureus* (MRSA) strains, reporting MIC values of 0.03906 mg/mL and MBC values ranging from 0.03906 to 0.07813 mg/mL. These values are highly consistent with those obtained in the present study, where the MIC and MBC for the reference *S. aureus* strain were 0.005% (*v*/*v*), corresponding to approximately 0.05 mg/mL. The lower susceptibility of Gram-negative bacteria is generally attributed to the presence of an outer membrane, which acts as an effective permeability barrier against many bioactive compounds [94,95]. The antibacterial activity observed in the present study is unlikely to result from a single constituent but rather from interactions among cannabinoids, terpenes, and other secondary metabolites present in the plant material. This observation is consistent with the concept of the *entourage effect*, according to which synergistic interactions among multiple constituents may produce greater biological activity than individual compounds administered separately. This interpretation is supported by the review of Schofs et al. [96], which summarizes current evidence on the antimicrobial activity of *Cannabis sativa*. The authors emphasized that most available studies are limited to the determination of MIC and MBC values, whereas the kinetics of bacterial killing assessed using time–kill assays have received considerably less attention. In the present study, the time–kill assay demonstrated that the extracts differed not only in their minimum inhibitory and bactericidal concentrations but also in the rate of bacterial killing. Complete elimination of *S. aureus* was achieved after only 2 h using inflorescence extracts from the Finola and Kompolti cultivars, whereas complete elimination of *E. coli* and *P. aeruginosa* required 8–12 h, depending on the extract tested. From a practical perspective, these findings indicate that the evaluation of antibacterial activity should not rely solely on MIC and MBC values but should also consider the kinetics of bactericidal activity, which may influence the efficacy of future topical antimicrobial formulations intended for veterinary use. Ensuring the microbiological safety of pet foods has become an increasingly important challenge due to the growing market share of products containing fresh animal-derived ingredients. In recent years, raw meat-based diets have been identified as potential sources of multidrug-resistant bacteria, including *Escherichia coli* and *Pseudomonas aeruginosa*, posing potential health risks to both companion animals and their owners [97,98]. In light of the present findings, hemp materials warrant further investigation as potential functional feed ingredients. In addition to their nutritional and phytochemical characteristics, their antibacterial activity may be relevant to the microbiological safety of pet foods. However, confirmation of this hypothesis requires studies conducted using complete pet food formulations and evaluation of their microbiological stability during processing and storage.

A strength of the present study is the comprehensive comparison of the chemical composition and in vitro antibacterial activity of leaves and inflorescences from four hemp cultivars. Nevertheless, several limitations should be acknowledged. The antibacterial activity was evaluated in vitro against only three reference bacterial strains, including *S. aureus* rather than *S. pseudintermedius*, which is more relevant to canine bacterial infections. Furthermore, the hemp materials were evaluated as individual raw materials and extracts and were not incorporated into complete dog food formulations. Therefore, the present results do not establish their efficacy in improving the microbiological safety of finished pet foods or their suitability as functional feed ingredients. Their digestibility, palatability, bioavailability, safety, and feeding efficacy also remain to be evaluated. Further studies using complete dog food formulations, additional bacterial strains, particularly *S. pseudintermedius*, and in vivo feeding trials are required to determine whether the properties observed in the present study translate into practical benefits in canine nutrition.

## 5. Conclusions

The present study demonstrated that hemp leaves and inflorescences differ markedly in their phytochemical composition, lipid quality, and antibacterial activity, suggesting that they should be regarded as complementary rather than interchangeable raw materials. Inflorescences contained significantly higher concentrations of cannabinoids and most terpenes, whereas leaves were characterized by a more favorable fatty acid profile, including higher proportions of n-3 polyunsaturated fatty acids, a lower n-6/n-3 ratio, and more favorable lipid quality indices (AI, TI, h/H, HPI, and DFA). These differences demonstrate distinct compositional characteristics of the two plant parts and provide a basis for further investigation of their potential nutritional and functional applications. In addition, cultivar significantly influenced the composition of cannabinoids, terpenes, and fatty acids, highlighting the importance of genotype selection in further studies evaluating hemp-derived materials for feed applications. All ethanolic extracts exhibited antibacterial activity against the tested bacterial strains, with the strongest effect observed against *Staphylococcus aureus.* Although the MIC and MBC values were identical for all extracts against *S. aureus* and *Escherichia coli*, the time–kill assay demonstrated considerable differences in the rate of bacterial elimination. The inflorescence extracts of the Finola and Kompolti cultivars showed the fastest bactericidal activity, highlighting the value of assessing antimicrobial properties should include both inhibitory concentrations and bacterial killing kinetics.

Overall, the results indicate that hemp leaves, despite being considerably less studied and often treated as an agricultural by-product, represent a valuable source of nutritionally relevant fatty acids and biologically active compounds, whereas inflorescences remain the richest source of cannabinoids and terpenes. Their complementary characteristics support further investigation of both plant parts as potential feed ingredients for companion animals while promoting more efficient utilization of hemp biomass within a circular bioeconomy. Future studies should focus on evaluating these raw materials in complete dog food formulations, including the effects of processing on phytochemical stability, the bioavailability of bioactive compounds, optimal inclusion levels, long-term safety, and their influence on animal health and the microbiological stability of finished pet foods.

## Figures and Tables

**Table 1:** Multiple reaction monitoring (MRM) transitions used for the identification and quantification of cannabinoids and isotopically labeled internal standards.

Analyte	Primary Ion	Secondary Ion (s)
CBD	315.2	135.1; 193.2
CBG	317.2	193.2; 123.1
CBN	311.2	223.2; 241.2
CBD-A	359.2	261.2; 233.2
CBC	315.2	259.2; 233.2
CBG-A	361.2	343.3; 219.2
THC-A	359.2	341.3; 273.2; 261.2
CBC-A	359.2	219.2; 285.2
D-CBD-A	362.2	222.1
D-CBG-A	364.3	222.3
D-THC-A	362.2	344.2
D-CBC-A	362.2	222.2

**Table 2:** Total cannabinoid content (mg/g DM) in raw materials of *Cannabis sativa* L.

Factor	Total Cannabinoids	Total CBC ^2^	Total CBD ^3^	Total CBG ^4^
Cultivar				
Finola	87.250 b ^1^	5.641 a	39.939 b	41.154 b
Futura 75	84.925 b	6.919 ab	74.806 c	2.368 a
Dioica	81.263 b	7.656 b	70.851 c	2.054 a
Kompolti	63.201 a	5.641 a	1.036 a	56.150 c
Plant part				
Inflorescences	115.740 b	8.461 b	69.110 b	37.465 b
Leaves	42.580 a	4.468 a	24.206 a	13.398 a

^1^ means with at least one same letter (a, b, c) not differ statistically at *p* = 0.05 (for all columns and factors separately); ^2^ CBC—cannabichromene; ^3^ CBD—cannabidiol; total CBD was calculated as the sum of CBD and CBD-A corrected by the molecular weight conversion factor (0.877); ^4^ CBG—cannabigerol; total CBG was calculated as the sum of CBG and CBG-A corrected by the molecular weight conversion factor (0.877).

**Table 3:** Individual cannabinoid content (mg/g DM) in raw materials of *Cannabis sativa* L.

Factor	CBC ^1^	CBC-A ^2^	CBD ^3^	CBD-A ^4^	CBG ^5^	CBG-A ^6^	CBN ^7^	THC-A ^8^
Cultivar								
Finola	1.469 b ^9^	4.758 a	8.044 b	36.368 b	2.813 b	43.719 b	0.031 b	0.553 b
Futura 75	1.081 a	6.657 b	10.174 c	73.697 c	0.525 a	2.102 a	0.049 d	0.893 d
Dioica	1.444 b	7.083 b	10.551 c	68.760 c	0.470 a	1.806 a	0.036 c	0.758 c
Kompolti	2.469 c	3.617 a	0.260 a	0.885 a	5.669 c	57.561 c	0.026 a	0.397 a
Plant part								
Inflorescences	1.961 b	7.413 b	10.880 b	66.398 b	2.981 b	39.321 b	0.047 b	0.749 b
Leaves	1.271 a	3.644 a	3.636 a	23.456 a	1.758 a	13.273 a	0.024 a	0.552 a

^1^ CBC—cannabichromene; ^2^ CBC-A—cannabichromenic acid; ^3^ CBD—cannabidiol; ^4^ CBD-A—cannabidiolic acid; ^5^ CBG—cannabigerol; ^6^ CBG-A—cannabigerolic acid; ^7^ CBN—cannabinol; ^8^ THC-A—tetrahydrocannabinolic acid; ^9^ means with at least one same letter (a, b, c, d) do not differ statistically at *p* = 0.05 (for all columns and factors separately).

**Table 4:** Terpene concentrations (μg/g DM) in raw materials of *Cannabis sativa* L.

Factor	α-Humulene	Linalool	β-Myrcene	Terpinen	Limonene
Cultivar					
Finola	303.483 c ^1^	72.755 b	17.964 ab	13.995 a	8.869 a
Futura 75	173.154 a	73.573 b	24.483 b	12.090 a	11.196 ab
Dioica	280.804 bc	101.050 c	40.603 c	13.911 a	12.941 b
Kompolti	220.265 ab	28.865 a	13.395 a	11.982 a	8.793 a
Plant part					
Inflorescences	334.378 b	108.174 b	38.069 b	11.647 a	13.215 b
Leaves	154.475 a	29.947 a	10.154 a	14.342 b	7.685 a

^1^ means with at least one same letter (a, b, c) not differ statistically at *p* = 0.05 (for all columns and factors separately).

**Table 5:** Fatty acid composition (% of total fatty acids) in raw materials of *Cannabis sativa* L.

Factor	C12:0	C14:0	C16:0	C18:0	C16:1 n-7	C18:1 n-9	C18:2 n-6	C18:3 n-3
Cultivar								
Finola	0.688 a ^1^	0.691 a	18.499 a	5.123 a	1.163 a	2.784 a	15.021 b	44.533 d
Futura 75	0.750 ab	0.829 b	22.207 c	7.418 d	1.346 ab	3.797 b	16.144 c	33.449 a
Dioica	0.865 ab	1.081 c	22.345 c	6.373 c	1.794 c	2.694 a	16.240 c	35.793 b
Kompolti	0.951 b	0.918 b	20.679 b	6.034 b	1.579 bc	2.555 a	13.178 a	39.920 c
Plant part								
Inflorescences	0.601 a	0.803 a	21.098 a	6.458 b	1.214 a	4.009 b	19.715 b	31.799 a
Leaves	1.026 b	0.957 b	20.767 a	6.016 a	1.726 b	1.906 a	10.576 a	45.049 b

^1^ means with at least one same letter (a, b, c, d) do not differ statistically at *p* = 0.05 (for all columns and factors separately).

**Table 6:** Fatty acid groups (% of total fatty acids) and n-6/n-3 ratio in raw materials of *Cannabis sativa* L.

Factor	SFA	MUFA	PUFA	Σ n-6	Σ n-3	n-6/n-3
Cultivar						
Finola	36.499 a ^1^	3.947 a	59.554 d	15.021 b	44.533 d	0.374 b
Futura 75	45.264 c	5.143 b	49.593 a	16.144 c	33.449 a	0.509 c
Dioica	43.480 b	4.487 ab	52.033 b	16.240 c	35.793 b	0.500 c
Kompolti	42.769 b	4.134 a	53.097 c	13.178 a	39.920 c	0.355 a
Plant part						
Inflorescences	43.264 b	5.223 b	51.514 a	19.715 b	31.799 a	0.628 b
Leaves	40.742 a	3.633 a	55.625 b	10.576 a	45.049 b	0.241 a

^1^ means with at least one same letter (a, b, c, d) do not differ statistically at *p* = 0.05 (for all columns and factors separately).

**Table 7:** Lipid quality indices in raw materials of *Cannabis sativa* L.

Factor	DFA	OFA	AI	TI	h/H	HPI
Cultivar						
Finola	62.338 d ^1^	18.880 a	5.470 a	0.173 a	3.159 c	2.907 c
Futura 75	53.391 a	23.786 c	7.898 d	0.273 d	2.245 a	2.085 a
Dioica	54.727 b	24.292 c	6.860 c	0.252 c	2.263 a	2.061 a
Kompolti	55.653 c	22.548 b	6.476 b	0.214 b	2.469 b	2.263 b
Plant part						
Inflorescences	55.522 a	22.503 a	6.898 b	0.262 b	2.475 a	2.287 a
Leaves	57.531 b	22.750 a	6.454 a	0.194 a	2.593 b	2.371 b

^1^ means with at least one same letter (a, b, c, d) do not differ statistically at *p* = 0.05 (for all columns and factors separately).

**Table 8:** Minimum inhibitory concentration (MIC) and minimum bactericidal concentration (MBC) of hemp extracts against three reference bacterial strains.

Reference Strain		Hemp Extract.							
		Finola		Futura 75		Dioica		Kompolti	
		IF ^1^	L ^2^	IF	L	IF	L	IF	L
		% (*v*/*v*)							
*Staphylococcus aureus* ATCC 6538	MIC ^3^	0.005	0.005	0.005	0.005	0.005	0.005	0.005	0.005
	MBC ^4^								
*Escherichia coli* ATCC 11303	MIC	0.625	0.625	0.625	0.625	0.625	0.625	0.625	0.625
	MBC								
*Pseudomonas* *aeruginosa* PCM 2710	MIC	1.25	1.25	2.5	1.25	2.5	2.5	1.25	1.25
	MBC	1.25	2.5	2.5	2.5	2.5	2.5	1.25	2.5

^1^ IF—inflorescences; ^2^ L—leaves, ^3^ MIC—minimum inhibitory concentration; ^4^ MBC—minimum bactericidal concentration.

## Data Availability

The data supporting the findings of this study are included in the article. Additional raw data are available from the corresponding author upon reasonable request.

## Supplementary Materials

The following supporting information can be downloaded at: https://www.mdpi.com/article/10.3390/ani16172635/s1, Table S1. Quantitative calibration ranges, estimated limits of detection (LOD), and limits of quantification (LOQ) for the analyzed cannabinoids; Table S2. Quantitative calibration ranges, estimated limits of detection (LOD), and limits of quantification (LOQ) for the analyzed terpenes and terpenoids; Table S3. Linearity ranges, coefficients of determination (R^2^), and estimated limits of detection (LOD) and quantification (LOQ) for the analyzed fatty acids; Table S4. Variability of major fatty acid contents among sample-preparation/extraction technical replicates; Figure S1. Representative GC-FID chromatogram of the reference-standard mixture obtained using the HP-88 capillary column under the chromatographic conditions described in Section 2.3; Figure S2. CBC content in the leaves (L) and inflorescences (IF) of *Cannabis sativa* L. cultivars (F—Finola, FR—Futura 75, D—Dioica, K—Kompolti); Figure S3. CBC-A content in the leaves (L) and inflorescences (IF) of *Cannabis sativa* L. cultivars (F—Finola, FR—Futura 75, D—Dioica, K—Kompolti; Figure S4. CBD content in the leaves (L) and inflorescences (IF) of *Cannabis sativa* L. cultivars (F—Finola, FR—Futura 75, D—Dioica, K—Kompolti; Figure S5. CBD-A content in the leaves (L) and inflorescences (IF) of *Cannabis sativa* L. cultivars (F—Finola, FR—Futura 75, D—Dioica, K—Kompolti); Figure S6. CBG content in the leaves (L) and inflorescences (IF) of *Cannabis sativa* L. cultivars (F—Finola, FR—Futura 75, D—Dioica, K—Kompolti; Figure S7. CBG-A content in the leaves (L) and inflorescences (IF) of *Cannabis sativa* L. cultivars (F—Finola, FR—Futura 75, D—Dioica, K—Kompolti; Figure S8. CBN content in the leaves (L) and inflorescences (IF) of *Cannabis sativa* L. cultivars (F—Finola, FR—Futura 75, D—Dioica, K—Kompolti); Figure S9. THC-A content in the leaves (L) and inflorescences (IF) of *Cannabis sativa* L. cultivars (F—Finola, FR—Futura 75, D—Dioica, K—Kompolti); Figure S10. α-humulene content in the leaves (L) and inflorescences (IF) of *Cannabis sativa* L. cultivars (F—Finola, FR—Futura 75, D—Dioica, K—Kompolti; Figure S11. β-myrcene content in the leaves (L) and inflorescences (IF) of *Cannabis sativa* L. cultivars (F—Finola, FR—Futura 75, D—Dioica, K—Kompolti); Figure S12. Limonene content in the leaves (L) and inflorescences (IF) of *Cannabis sativa* L. cultivars (F—Finola, FR—Futura 75, D—Dioica, K—Kompolti; Figure S13. Linalool content in the leaves (L) and inflorescences (IF) of *Cannabis sativa* L. cultivars (F—Finola, FR—Futura 75, D—Dioica, K—Kompolti); Figure S14. Terpinen content in the leaves (L) and inflorescences (IF) of *Cannabis sativa* L. cultivars (F—Finola, FR—Futura 75, D—Dioica, K—Kompolti).

## Abbreviations

The following abbreviations are used in this manuscript:

AI	Index of Atherogenicity
ALA	Alpha-linolenic acid
ALP	Alkaline phosphatase
ALT	Alanine aminotransferase
AST	Aspartate aminotransferase
CBC	Cannabichromene
CBD	Cannabidiol
CBD-A	Cannabidiolic acid
CBG	Cannabigerol
CBG-A	Cannabigerolic acid
CBN	Cannabinol
DFA	Desirable fatty acids
DM	Dry matter
DMSO	Dimethyl sulfoxide
EFA	Essential fatty acids
HPI	Health-Promoting Index
h/H	Hypocholesterolemic-to-hypercholesterolemic fatty acid ratio
IL-1β	Interleukin 1 beta
IL-6	Interleukin 6
LA	Linoleic acid
MBC	Minimum bactericidal concentration
MHB	Mueller–Hinton broth
MIC	Minimum inhibitory concentration
MRSA	Methicillin-resistant *Staphylococcus aureus*
MRSP	Methicillin-resistant *Staphylococcus pseudintermedius*
MUFA	Monounsaturated fatty acids
NF-κB	Nuclear factor kappa B
OFA	Undesirable fatty acids
PUFA	Polyunsaturated fatty acids
SFA	Saturated fatty acids
Δ9-THC	Delta-9-tetrahydrocannabinol
THC	Tetrahydrocannabinol
THC-A	Tetrahydrocannabinolic acid
TI	Index of Thrombogenicity
TNF-α	Tumor necrosis factor alpha
UFA	Unsaturated fatty acids

## References

[1] Knight A. (2026). Sustainable pet diets: A leading effective altruism issue. Animals.

[2] Kazemi M. (2025). Algae as a sustainable feed resource: Revolutionizing animal nutrition. Aquac. Int..

[3] Kaur G., Kander R. (2023). The sustainability of industrial hemp: A literature review of its economic, environmental, and social sustainability. Sustainability.

[4] de Oliveira C.T., Oliveira G.G.A. (2023). What circular economy indicators really measure? An overview of circular economy principles and sustainable development goals. Resour. Conserv. Recycl..

[5] Velenturf A.P.M., Purnell P. (2021). Principles for a sustainable circular economy. Sustain. Prod. Consum..

[6] Karche T., Singh M.R. (2019). The application of hemp (*Cannabis sativa* L.) for a green economy: A review. Turk. J. Bot..

[7] Hancz C., Sultana S., Nagy Z., Biró J. (2024). The role of insects in sustainable animal feed production for environmentally friendly agriculture: A review. Animals.

[8] Nielson S.A., Khosa D.K., Clow K.M., Verbrugghe A. (2023). Dog caregivers’ perceptions, motivations, and behaviours for feeding treats: A cross-sectional study. Prev. Vet. Med..

[9] Bartončíková M., Lapčíková B., Lapčík L., Valenta T. (2023). Hemp-derived CBD used in food and food supplements. Molecules.

[10] Xu J., Bai M., Song H., Yang L., Zhu D., Liu H. (2022). Hemp (*Cannabis sativa* subsp. sativa) chemical composition and the application of hempseeds in food formulations. Plant Foods Hum. Nutr..

[11] Grigoryev S.V., Grigoryev S.V., Illarionova K.V., Shelenga T.V., Vavilov N.I. (2020). Hempseeds (*Cannabis* spp.) as a source of functional food ingredients, prebiotics and phytosterols. Agric. Food Sci..

[12] Tănase Apetroaei V., Pricop E.M., Istrati D.I., Vizireanu C. (2024). Hemp seeds (*Cannabis sativa* L.) as a valuable source of natural ingredients for functional foods—A review. Molecules.

[13] Rizzo G., Storz M.A., Calapai G. (2023). The role of hemp (*Cannabis sativa* L.) as a functional food in vegetarian nutrition. Foods.

[14] Giupponi L., Leoni V., Pavlovic R., Giorgi A. (2020). Influence of altitude on phytochemical composition of hemp inflorescence: A metabolomic approach. Molecules.

[15] Jokić S., Jerković I., Pavić V., Aladić K., Molnar M., Kovač M.J., BRAK (2022). Terpenes and cannabinoids in supercritical CO_2_ extracts of industrial hemp inflorescences: Optimization of extraction, antiradical and antibacterial activity. Pharmaceuticals.

[16] Jin D., Dai K., Xie Z., Chen J. (2020). Secondary metabolites profiled in Cannabis inflorescences, leaves, stem barks, and roots for medicinal purposes. Sci. Rep..

[17] Beleggia R., Menga V., Fulvio F., Fares C., Trono D. (2023). Effect of genotype, year, and their interaction on the accumulation of bioactive compounds and the antioxidant activity in industrial hemp (*Cannabis sativa* L.) inflorescences. Int. J. Mol. Sci..

[18] Knezevic F., Nikolai A., Marchart R., Sosa S., Tubaro A., Novak J. (2021). Residues of herbal hemp leaf teas—How much of the cannabinoids remain?. Food Control.

[19] Galanty A., Juncewicz P., Podolak I., Grabowska K., Służały P., Paśko P. (2024). Comparative analysis of polyphenolic profile and chemopreventive potential of hemp sprouts, leaves, and flowers of the Sofia variety. Plants.

[20] Frusciante L., Geminiani M., Shabab B., Olmastroni T., Roncucci N., Mastroeni P., Salvini L., Lamponi S., Trezza A., Santucci A. (2025). Enhancing industrial hemp (*Cannabis sativa*) leaf by-products: Bioactive compounds, anti-inflammatory properties, and potential health applications. Int. J. Mol. Sci..

[21] Zhang L., Gao F., Ge J., Li H., Xia F., Bai H., Piao X., Shi L. (2022). Potential of aromatic plant-derived essential oils for the control of foodborne bacteria and antibiotic resistance in animal production: A review. Antibiotics.

[22] Kačániová M., Galovičová L., Borotová P., Valková V., Ďúranová H., Kowalczewski P.Ł., Said-Al Ahl H.A.H., Hikal W.M., Vukic M., Savitskaya T. (2021). Chemical composition, in vitro and in situ antimicrobial and antibiofilm activities of *Syzygium aromaticum* (clove) essential oil. Plants.

[23] de Morais Oliveira-Tintino C.D., Tintino S.R., Limaverde P.W., Figueredo F.G., Campina F.F., da Cunha F.A.B., da Costa R.H.S., Pereira P.S., Lima L.F., de Matos Y.M.L. (2018). Inhibition of the essential oil from *Chenopodium ambrosioides* L. and α-terpinene on the NorA efflux-pump of *Staphylococcus aureus*. Food Chem..

[24] Silva V.A., Sousa J.P., de Luna Freire Pessôa H., de Freitas A.A.R., Coutinho H.D.M., Alves L.B.N., Lima E.O. (2016). *Ocimum basilicum*: Antibacterial activity and association study with antibiotics against bacteria of clinical importance. Pharm. Biol..

[25] Pontes E.K.U., Melo H.M., Nogueira J.W.A., Firmino N.C.S., de Carvalho M.G., Catunda Júnior F.E.A., Cavalcante T.T.A. (2019). Antibiofilm activity of the essential oil of citronella (*Cymbopogon nardus*) and its major component, geraniol, on the bacterial biofilms of *Staphylococcus aureus*. Food Sci. Biotechnol..

[26] Mehdi Y., Létourneau-Montminy M.P., Gaucher M.L., Chorfi Y., Suresh G., Rouissi T., Brar S.K., Côté C., Ramirez A.A., Godbout S. (2018). Use of antibiotics in broiler production: Global impacts and alternatives. Anim. Nutr..

[27] Pieracci Y., Ascrizzi R., Terreni V., Pistelli L., Flamini G., Bassolino L., Fulvio F., Montanari M., Paris R. (2021). Essential oil of *Cannabis sativa* L.: Comparison of yield and chemical composition of 11 hemp genotypes. Molecules.

[28] Araújo M., Almeida M.B., Araújo L.L.N. (2023). The cannabinoids mechanism of action: An overview. Braz. J. Pain.

[29] Salam U., Ullah S., Tang Z.-H., Elateeq A.A., Khan Y., Khan J., Khan A., Ali S. (2023). Plant metabolomics: An overview of the role of primary and secondary metabolites against different environmental stress factors. Life.

[30] Delgoda R., Murray J.E., Badal S., Delgoda R. (2017). Evolutionary perspectives on the role of plant secondary metabolites. Pharmacognosy: Fundamentals, Applications and Strategies.

[31] Samtiya M., Aluko R.E., Dhewa T., Moreno-Rojas J.M. (2021). Potential health benefits of plant food-derived bioactive components: An overview. Foods.

[32] Šaponjac V.T., Čanadanović-Brunet J., Ćetković G., Djilas S., Nedović V., Raspor P., Lević J., Šaponjac V.T., Barbosa-Cánovas G.V. (2016). Detection of bioactive compounds in plants and food products. Emerging and Traditional Technologies for Safe, Healthy and Quality Food.

[33] Journal of Laws of the Republic of Poland (2022). Act of 24 March 2022 Amending the Act on Counteracting Drug Addiction (Journal of Laws 2022, Item 763).

[34] Izzo L., Pacifico S., Piccolella S., Castaldo L., Narváez A., Grosso M., Ritieni A. (2020). Chemical analysis of minor bioactive components and cannabidiolic acid in commercial hemp seed oil. Molecules.

[35] Da Porto C., Decorti D., Tubaro F. (2012). Fatty acid composition and oxidation stability of hemp (*Cannabis sativa* L.) seed oil extracted by supercritical carbon dioxide. Ind. Crops Prod..

[36] Irakli M., Tsaliki E., Kalivas A., Kleisiaris F., Sarrou E., Cook C.M. (2019). Effect of genotype and growing year on the nutritional, phytochemical, and antioxidant properties of industrial hemp (*Cannabis sativa* L.) seeds. Antioxidants.

[37] Abedi E., Sahari M.A. (2014). Long-chain polyunsaturated fatty acid sources and evaluation of their nutritional and functional properties. Food Sci. Nutr..

[38] Sokoła-Wysoczańska E., Wysoczański T., Wagner J., Czyż K., Bodkowski R., Lochyński S., Patkowska-Sokoła B. (2018). Polyunsaturated fatty acids and their potential therapeutic role in cardiovascular system disorders—A review. Nutrients.

[39] Schlotter Y.M., Rutten V., Riemers F., Davenport G., Knol E.F., Willemse T. (2009). Altered expression of fatty acid desaturases in the skin of dogs with atopic dermatitis. J. Dermatol. Sci..

[40] Logas D., Kunkle G.A. (1994). Double-blinded crossover study with marine oil supplementation containing high-dose eicosapentaenoic acid for the treatment of canine pruritic skin disease. Vet. Dermatol..

[41] Sorokin A.V., Arnardottir H., Svirydava M., Ng Q., Baumer Y., Berg A., Pantoja C.J., Florida E.M., Teague H.L., Yang Z.-H. (2023). Comparison of the dietary omega-3 fatty acids impact on murine psoriasis-like skin inflammation and associated lipid dysfunction. J. Nutr. Biochem..

[42] Czumaj A., Śledziński T. (2020). Biological role of unsaturated fatty acid desaturases in health and disease. Nutrients.

[43] Combarros D., Castilla-Castaño E., Lecru L.A., Pressanti C., Amalric N., Cadiergues M.C. (2020). A prospective, randomized, double blind, placebo-controlled evaluation of the effects of an n-3 essential fatty acids supplement (Agepi^®^ ω_3_) on clinical signs, and fatty acid concentrations in the erythrocyte membrane, hair shafts and skin surface of dogs with poor quality coats. Prostaglandins Leukot. Essent. Fat. Acids.

[44] Stehle M.E., Hanczaruk M., Schwarz S.C.N., Göbel T.W., Mueller R.S. (2010). Effects of polyunsaturated fatty acids on isolated canine peripheral blood mononuclear cells and cytokine expression (IL-4, IFN-γ, TGF-β) in healthy and atopic dogs. Vet. Dermatol..

[45] de Santiago M.S., Arribas J.L.G., Llamas Y.M., Becvarova I., Meyer H. (2021). Randomized, double-blind, placebo-controlled clinical trial measuring the effect of a dietetic food on dermatologic scoring and pruritus in dogs with atopic dermatitis. BMC Vet. Res..

[46] Di Giacomo V., Recinella L., Chiavaroli A., Orlando G., Cataldi A., Rapino M., Di Valerio V., Politi M., Antolini M.D., Acquaviva A. (2021). Metabolomic profile and antioxidant/anti-inflammatory effects of industrial hemp water extract in fibroblasts, keratinocytes and isolated mouse skin specimens. Antioxidants.

[47] Huang S., Li H., Xu J., Zhou H., Seeram N.P., Ma H., Gu Q. (2023). Chemical constituents of industrial hemp roots and their anti-inflammatory activities. J. Cannabis Res..

[48] Jimenez A.G., Winward J., Beattie U., Cipolli W. (2018). Cellular metabolism and oxidative stress as a possible determinant for longevity in small breed and large breed dogs. PLoS ONE.

[49] Muršec A., Poljšak B., Nemec Svete A., Erjavec V. (2025). Antioxidant strategies for age-related oxidative damage in dogs. Vet. Sci..

[50] Ghosh N., Das A., Chaffee S., Roy S., Sen C.K., Watson R.R., Zibadi S., Preedy V.R. (2018). Reactive oxygen species, oxidative damage and cell death. Immunity and Inflammation in Health and Disease: Emerging Roles of Nutraceuticals and Functional Foods in Immune Support.

[51] Blanca P.M., María Luisa F.R., Guadalupe M., Fátima C.L. (2024). Oxidative stress in canine diseases: A comprehensive review. Antioxidants.

[52] Juodžentė D., Karvelienė B., Riškevičienė V. (2018). The influence of the duration of the preoperative time spent in the veterinary clinic without the owner on the psychogenic and oxidative stress in dogs. J. Vet. Med. Sci..

[53] Reimann M.J., Häggström J., Møller J.E., Lykkesfeldt J., Falk T., Olsen L.H. (2017). Markers of oxidative stress in dogs with myxomatous mitral valve disease are influenced by sex, neuter status, and serum cholesterol concentration. J. Vet. Intern. Med..

[54] Kapun A.P., Salobir J., Levart A., Kotnik T., Svete A.N. (2012). Oxidative stress markers in canine atopic dermatitis. Res. Vet. Sci..

[55] Passantino A., Quartarone V., Pediliggeri M.C., Rizzo M., Piccione G. (2014). Possible application of oxidative stress parameters for the evaluation of animal welfare in sheltered dogs subjected to different environmental and health conditions. J. Vet. Behav..

[56] Zych S., Adaszyńska-Skwirzyńska M., Szewczuk M.A., Szczerbińska D. (2024). Interaction between enrofloxacin and three essential oils (cinnamon bark, clove bud and lavender flower)—A study on multidrug-resistant *Escherichia coli* strains isolated from 1-day-old broiler chickens. Int. J. Mol. Sci..

[57] Jubair N., Rajagopal M., Chinnappan S., Abdullah N.B., Fatima A. (2021). Review on the antibacterial mechanism of plant-derived compounds against multidrug-resistant bacteria (MDR). Evid. Based Complement. Altern. Med..

[58] Jeong J.Y., Jung I.G., Yum S.H., Hwang Y.J. (2023). In vitro synergistic inhibitory effects of plant extract combinations on bacterial growth of methicillin-resistant *Staphylococcus aureus*. Pharmaceuticals.

[59] Techaoei S. (2022). Time-kill kinetics and antimicrobial activities of Thai medical plant extracts against fish pathogenic bacteria. J. Adv. Pharm. Technol. Res..

[60] Vaou N., Stavropoulou E., Voidarou C., Tsigalou C., Bezirtzoglou E. (2021). Towards advances in medicinal plant antimicrobial activity: A review study on challenges and future perspectives. Microorganisms.

[61] Park S.-H., Pauli C.S., Gostin E.L., Staples S.K., Seifried D., Kinney C., Vanden Heuvel B.D. (2022). Effects of short-term environmental stresses on the onset of cannabinoid production in young immature flowers of industrial hemp (*Cannabis sativa* L.). J. Cannabis Res..

[62] Stack G.M., Toth J.A., Carlson C.H., Cala A.R., Marrero-González M.I., Wilk R.L., Gentner D.R., Crawford J.L., Philippe G., Rose J.K.C. (2021). Season-long characterization of high-cannabinoid hemp (*Cannabis sativa* L.) reveals variation in cannabinoid accumulation, flowering time, and disease resistance. GCB Bioenergy.

[63] Pavlovic R., Panseri S., Giupponi L., Leoni V., Citti C., Cattaneo C., Cavaletto M., Giorgi A. (2019). Phytochemical and ecological analysis of two varieties of hemp (*Cannabis sativa* L.) grown in a mountain environment of Italian Alps. Front. Plant Sci..

[64] Francisco V.P., Cerny M., Valentin R., Milone-Delacourt F., Paillard A., Alignan M. (2024). Development of GC–MS coupled to GC–FID method for the quantification of cannabis terpenes and terpenoids: Application to the analysis of five commercial varieties of medicinal cannabis. J. Chromatogr. B.

[65] Addo P.W., Sagili S.U.K.R., Bilodeau S.E., Gladu-Gallant F.-A., MacKenzie D.A., Bates J., McRae G., MacPherson S., Paris M., Raghavan V. (2022). Cold ethanol extraction of cannabinoids and terpenes from cannabis using response surface methodology: Optimization and comparative study. Molecules.

[66] Sagili S.U.K.R., Addo P.W., MacPherson S., Shearer M., Taylor N., Paris M., Lefsrud M., Orsat V. (2023). Effects of particle size, solvent type, and extraction temperature on the extraction of crude cannabis oil, cannabinoids, and terpenes. ACS Food Sci. Technol..

[67] Ulbricht T.L.V., Southgate D.A.T. (1991). Coronary heart disease: Seven dietary factors. Lancet.

[68] Santos-Silva J., Bessa R., Santos-Silva F. (2002). Effect of genotype, feeding system and slaughter weight on the quality of light lambs. II. Fatty acid composition of meat. Livest. Prod. Sci..

[69] Chen S., Bobe G., Zimmerman S., Hammond E.G., Luhman C.M., Boylston T.D., Freeman A.E., Beitz D.C. (2004). Physical and sensory properties of dairy products from cows with various milk fatty acid compositions. J. Agric. Food Chem..

[70] Chen J., Liu H. (2020). Nutritional indices for assessing fatty acids: A mini-review. Int. J. Mol. Sci..

[71] André R., Gomes A.P., Pereira-Leite C., Marques-da-Costa A., Monteiro Rodrigues L., Sassano M., Rijo P., Costa M.C. (2024). The entourage effect in cannabis medicinal products: A comprehensive review. Pharmaceuticals.

[72] Al-Khazaleh A.K., Zhou X., Bhuyan D.J., Münch G.W., Al-Dalabeeh E.A., Jaye K., Chang D. (2024). The neurotherapeutic arsenal in *Cannabis sativa*: Insights into anti-neuroinflammatory and neuroprotective activity and potential entourage effects. Molecules.

[73] Di Salvo A., Conti M.B., della Rocca G. (2023). Pharmacokinetics, efficacy, and safety of cannabidiol in dogs: An update of current knowledge. Front. Vet. Sci..

[74] Kogan L.R., Hellyer P.W., Downing R. (2020). The use of cannabidiol-rich hemp oil extract to treat canine osteoarthritis-related pain: A pilot study. AHVMA J..

[75] Garcia G.A., Kube S., Carrera-Justiz S., Tittle D., Wakshlag J.J. (2022). Safety and efficacy of cannabidiol-cannabidiolic acid rich hemp extract in the treatment of refractory epileptic seizures in dogs. Front. Vet. Sci..

[76] Kosukwatthana P., Rungsuriyawiboon O., Rattanasrisomporn J., Kimram K., Tansakul N. (2024). Cytotoxicity and immunomodulatory effects of cannabidiol on canine PBMCs: A study in LPS-stimulated and epileptic dogs. Animals.

[77] Miranda-Cortés A., Mota-Rojas D., Crosignani-Outeda N., Casas-Alvarado A., Martínez-Burnes J., Olmos-Hernández A., Mora-Medina P., Verduzco-Mendoza A., Hernández-Ávalos I. (2023). The role of cannabinoids in pain modulation in companion animals. Front. Vet. Sci..

[78] Wakshlag J.J., Schwark W.S., Deabold K.A., Talsma B.N., Cital S., Lyubimov A., Iqbal A., Zakharov A. (2020). Pharmacokinetics of cannabidiol, cannabidiolic acid, Δ9-tetrahydrocannabinol, tetrahydrocannabinolic acid and related metabolites in canine serum after dosing with three oral forms of hemp extract. Front. Vet. Sci..

[79] Hanuš L.O., Hod Y. (2020). Terpenes/terpenoids in *Cannabis*: Are they important?. Med. Cannabis Cannabinoids.

[80] Booth J.K., Page J.E., Bohlmann J. (2017). Terpene synthases from *Cannabis sativa*. PLoS ONE.

[81] Radwan M.M., Chandra S., Gul S., ElSohly M.A. (2021). Cannabinoids, phenolics, terpenes and alkaloids of *Cannabis*. Molecules.

[82] Bensignor E., Morgan D.M., Nuttall T. (2008). Efficacy of an essential fatty acid-enriched diet in managing canine atopic dermatitis: A randomized, single-blinded, cross-over study. Vet. Dermatol..

[83] Popović T., Debeljak Martačić J., Pokimica B., Ravić B., Ranković S., Glibetić M., Stepanović P. (2023). Phospholipid fatty acid profiles of plasma and erythrocyte membranes in dogs fed with commercial granulated food. Acta Vet. Brno.

[84] Hensel P., Santoro D., Favrot C., Hill P., Griffin C. (2015). Canine atopic dermatitis: Detailed guidelines for diagnosis and allergen identification. BMC Vet. Res..

[85] Mueller R., Fieseler K., Rosychuk R., Ogilvie G., Greenwalt T. (2004). Effect of omega-3 fatty acids on canine atopic dermatitis. J. Small Anim. Pract..

[86] Banton S., Pezzali J.G., Richards T., Hillyer L.M., Ma D.W.L., Pisco J.M., Templeman J.R., Shoveller A.K. (2025). Feeding of a high protein, low carbohydrate diet leads to greater postprandial energy expenditure and fasted n6:n3 fatty acid ratio in lean, adult dogs compared to a moderate protein, moderate carbohydrate diet. Transl. Anim. Sci..

[87] Leiva A., Granados-Chinchilla F. (2020). Fatty acid profiling in animal feeds and related food matrixes using a fast GC/MS method and in situ derivatization. Int. J. Agric. Environ. Food Sci..

[88] Portolesi R., Powell B.C., Gibson R.A. (2007). Competition between 24:5n-3 and ALA for Δ6 desaturase may limit the accumulation of DHA in HepG2 cell membranes. J. Lipid Res..

[89] Valenzuela R., Metherel A.H., Cisbani G., Smith M.E., Chouinard-Watkins R., Klievik B.J., Farias C., Videla L.A., Bazinet R.P. (2025). Specific activity of mouse liver desaturases and elongases: Time course effects using n-3 and n-6 PUFA substrates and inhibitory responses of delta-6 desaturase. Biochim. Biophys. Acta Mol. Cell Biol. Lipids.

[90] Vastolo A., Iliano S., Laperuta F., Pennacchio S., Pompameo M., Cutrignelli M.I. (2021). Hemp seed cake as a novel ingredient for dog’s diet. Front. Vet. Sci..

[91] Kępińska-Pacelik J., Biel W., Witkowicz R., Micek P., Piątkowska E., Patla A. (2025). Changes in the fatty acid composition and antioxidant properties in mono-protein commercial dry dog foods during storage. Molecules.

[92] Bannoehr J., Guardabassi L. (2012). *Staphylococcus pseudintermedius* in the dog: Taxonomy, diagnostics, ecology, epidemiology and pathogenicity. Vet. Dermatol..

[93] Carroll K.C., Burnham C.A.D., Westblade L.F. (2021). From canines to humans: Clinical importance of *Staphylococcus pseudintermedius*. PLoS Pathog..

[94] Ali E.M.M., Almagboul A.Z.I., Khogali S.M.E., Gergeir U.M.A. (2012). Antimicrobial activity of *Cannabis sativa* L. Chin. Med..

[95] Muscarà C., Smeriglio A., Trombetta D., Mandalari G., La Camera E., Occhiuto C., Grassi G., Circosta C. (2021). Antioxidant and antimicrobial activity of two standardized extracts from a new Chinese accession of non-psychotropic *Cannabis sativa* L. Phytother. Res..

[96] Schofs L., Sparo M.D., Sánchez Bruni S.F. (2021). The antimicrobial effect behind *Cannabis sativa*. Pharmacol. Res. Perspect..

[97] Morgan G., Williams N., Llewelyn J., Haldenby S., Pinchbeck G., Timofte D. (2026). Isolation of carbapenem-resistant *Pseudomonas aeruginosa* and other Gram-negative ESKAPE organisms from samples of raw-meat diets for dogs in the UK. Vet. Rec..

[98] Antunes P., Novais C., Peixe L., Freitas A.R. (2024). Pet food safety: Emerging bacterial hazards and implications for public health. Curr. Opin. Food Sci..

