# Supplementary material for: Bioactive Compounds and In Vitro Antibacterial Activity of Hemp Leaves and Inflorescences as Potential Functional Feed Ingredients for Canine Nutrition
Source: Animals (Basel). 2026 Aug 22;16(17):2635. doi: 10.3390/ani16172635 (PMC13564278; doi:10.3390/ani16172635)
Supplement: Supplementary file 1 [file animals-16-02635-s001.zip › animals-4512499-supplementary.pdf]

Table S1. Quantitative calibration ranges, estimated limits of detection (LOD), and limits of quantification (LOQ) for the analyzed cannabinoids.

Analyte	Quantitative calibration range (μg/mL)[1]	S/N reference concentration for LOD (μg/mL)	S/N at reference concentration	Estimated LOD (μg/mL) (S/N = 3)[2]	LOQ (μg/mL) (lowest standard with S/N ≥ 10)[3]	S/N at LOQ
CBC	1–300	0.500	7.90	0.190	1.000	14.35
CBC-A	20–300	5.000	4.00	3.750	20.000	17.20
CBD	1–300	0.500	5.60	0.268	1.000	13.15
CBD-A	0.5–300	0.500	13.72	0.109	0.500	13.72
CBG	0.5–300	0.500	64.35	0.023	0.500	64.35
CBG-A	0.5–300	0.500	108.93	0,014	0.500	108.93
CBN	0.25–5	0.250	23.24	0.032	0.250	23.24
THC-A	0.5–50	0.500	11.70	0.128	0.500	11.70

[1]For CBD and CBC, the 0.5 μg/mL standards were detectable but provided S/N < 10; therefore, 1 μg/mL was used as the lower limit of the quantitative range, for CBC-A, the 5 μg/mL standard provided S/N = 4.0, whereas the 20 μg/mL standard provided S/N = 17.2; therefore, 20 μg/mL was used as the lower limit of the quantitative range; [2]LOD was estimated as C_ref × 3/(S/N), where C_ref is the concentration of the low-concentration reference standard; [3]LOQ was defined as the lowest experimentally measured calibration standard providing S/N ≥ 10 and was not mathematically extrapolated. LOD and LOQ values represent instrumental estimates based on calibration standards and should not be interpreted as full matrix-validated method LOD and LOQ values.

Table S2. Quantitative calibration ranges, estimated limits of detection (LOD), and limits of quantification (LOQ) for the analyzed terpenes and terpenoids.

Analyte	Quantitative calibration range (μg/mL)[1]	S/N reference concentration for LOD (μg/mL)	S/N at reference concentration	Estimated LOD (μg/mL) (S/N = 3)[2]	LOQ (μg/mL) (lowest standard with S/N ≥ 10)[3]	S/N at LOQ
α-Humulene	0.390625–12.5	0.390625	21.00	0.0558	0.390625	21.00
Linalool	0.1953–6.25	0.195313	15.80	0.0371	0.195313	15.80
β-Myrcene	0.0977–1.5625	0.097656	10.10	0.0290	0.097656	10.10
Terpinene	0.78125–6.25	0.781250	11.70	0.2003	0.781250	11.70
Limonene	0.0977–1.5625	0.097656	15.80	0.0185	0.097656	15.80

[1]Calibration standards were prepared as an 11-point two-fold serial dilution covering 0.0977–100 μg/mL; for each analyte, the regression range was restricted to points encompassing concentrations observed in the analyzed samples and meeting the quantification criterion. Quantification was performed by external calibration using authentic reference standards; no internal standards were used. LOD and LOQ values represent instrumental estimates based on calibration standards rather than full matrix-validated method values; [2]LOD was estimated as C_ref × 3/(S/N), where C_ref is the concentration of the low-concentration reference standard; [3]LOQ was defined as the lowest experimentally measured calibration standard providing S/N ≥ 10 and was not mathematically extrapolated.

Table S3. Linearity ranges, coefficients of determination (R^2), and estimated limits of detection (LOD) and quantification (LOQ) for the analyzed fatty acids.

Analyte	L0–L7 linearity range (μg/mL)[1]	L6 concentrati on (μg/mL)[2]	S/N at L6	Estimated LOD (μg/mL) (S/N = 3)[3]	Estimated LOQ (μg/mL) (S/N = 10)[3]	R^2 (L0–L7)[4]	L0 concentration (μg/mL)
C12:0	3.1172–399	6.2344	55.7	0.336	1.119	0.9982	399.0
C14:0	3.0938–396	6.1875	60.5	0.307	1.023	0.9980	396.0

C16:0	4.6875–600	9.3750	105.0	0.268	0.893	0.9990	600.0
C18:0	3.1172–399	6.2344	85.5	0.219	0.729	0.9984	399.0
C16:1 n-7	1.5469–198	3.0938	39.0	0.238	0.793	0.9956	198.0
C18:1 n-9	3.1172–399	6.2344	97.2	0.192	0.641	0.9989	399.0
C18:2 n-6	1.5469–198	3.0938	40.1	0.231	0.772	0.9949	198.0
C18:3 n-3	1.5469–198	3.0938	95.6	0.097	0.324	0.9965	198.0

[1]Linearity was assessed using the Supelco 37 Component FAME Mix and an eight-point two-fold serial dilution series (L0–L7), corresponding to a 128-fold concentration range. An additional L8 dilution was prepared but excluded from regression because it was below the useful quantitative response range for several analytes. The fatty-acid composition reported in the manuscript is expressed as relative content (% of total identified fatty acids), rather than as absolute concentrations. These calibration data were used to verify detector-response linearity and analytical sensitivity rather than for absolute quantification of FAME concentrations in the samples; [2]Signal-to-noise ratios were determined at level L6 (64-fold dilution); [3]LOD and LOQ were estimated as C(L6) × 3/(S/N) and C(L6) × 10/(S/N), respectively; [4]Linear regression was unweighted. R^2 values for L0–L7 ranged from 0.9949 to 0.9990.

Table S4. Variability of major fatty acid contents among sample-preparation/extraction technical replicates.

Fatty acid	Mean RSD across biological triplicates[1]	Range of RSD[2]
C16:0	2.07%	0.39–3.59%
C18:0	2.44%	1.56–4.24%
C18:2 n-6	1.69%	0.55–5.61%
C18:3 n-3	1.05%	0.08–1.97%

[1]RSDs were calculated from three independently weighed sample-preparation/extraction technical replicates (n = 3) obtained from the same pooled plant material for each cultivar × plant-part combination. The mean RSD represents the average of the RSD values obtained across all cultivar × plant-part combinations; [2]The RSD range represents the minimum and maximum RSD values observed across the cultivar × plant-part combinations. The reported variability reflects variability associated with sample preparation, extraction, transmethylation, and instrumental analysis and does not represent biological variability or formal analytical repeatability.

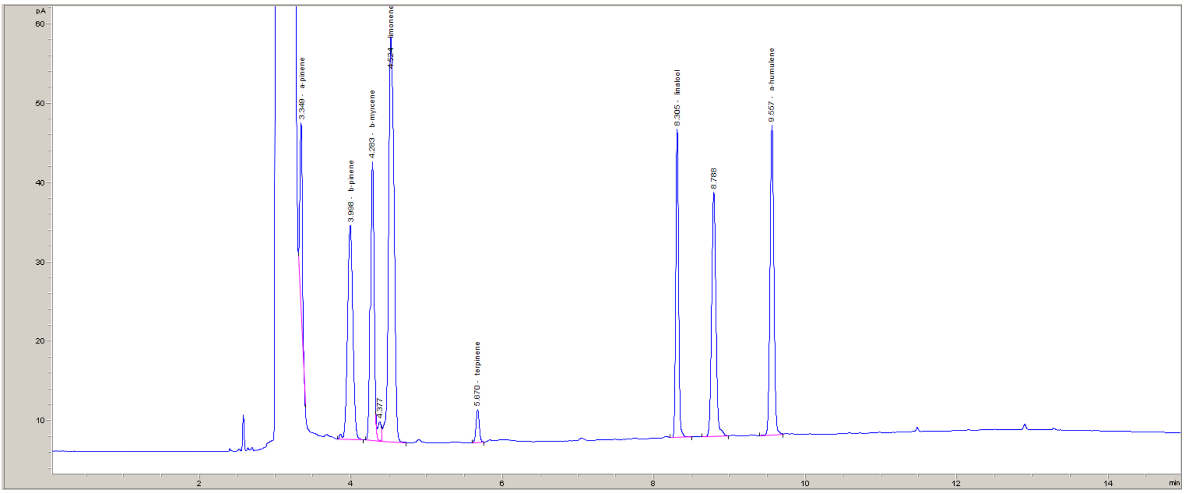

Figure S1. Representative GC-FID chromatogram of the reference-standard mixture obtained using the HP-88 capillary column under the chromatographic conditions described in Section 2.3. The

compounds quantified in the study were identified by comparison of their retention times with those of authentic reference standards.

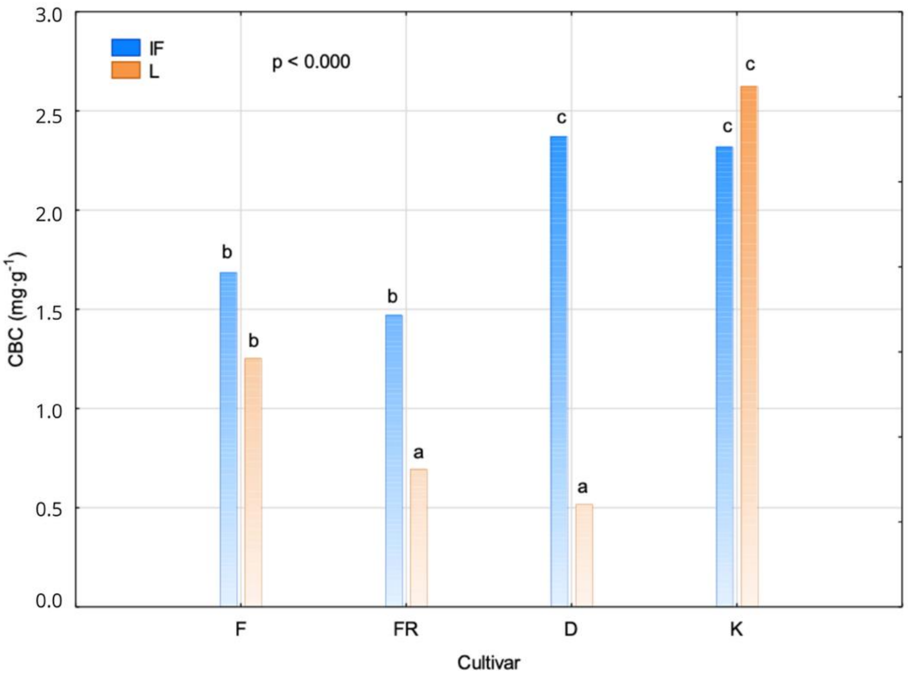

Figure S2. CBC content in the leaves (L) and inflorescences (IF) of *Cannabis sativa* L. cultivars (F—Finola, FR—Futura 75, D—Dioica, K—Kompolti). Data are presented as mean values. Different letters above the bars indicate significant differences among the analyzed groups (Tukey's HSD test, $p < 0.000$).

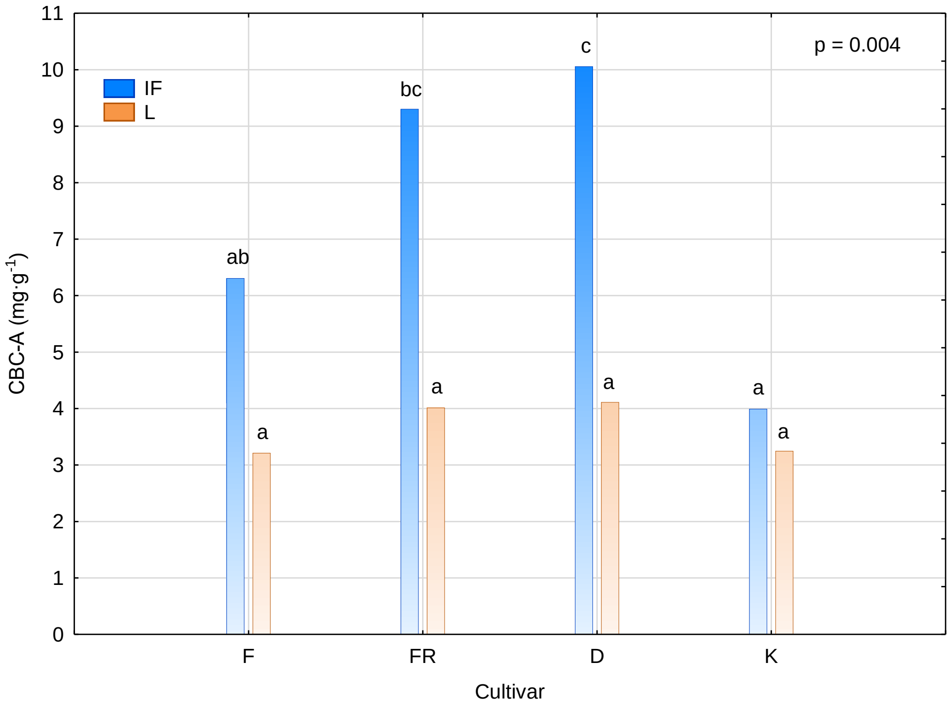

Figure S3. CBC-A content in the leaves (L) and inflorescences (IF) of *Cannabis sativa* L. cultivars (F—Finola, FR—Futura 75, D—Dioica, K—Kompolti). Data are presented as mean values, different letters above the bars indicate significant differences among the analyzed groups (Tukey's HSD test, $p < 0.004$).

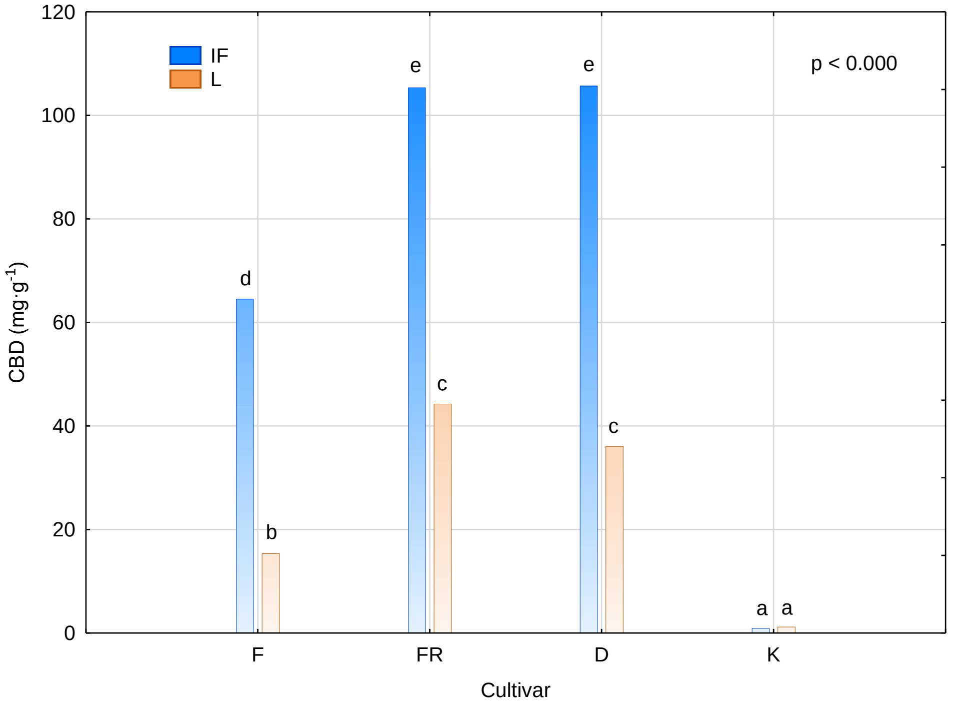

Figure S4. CBD content in the leaves (L) and inflorescences (IF) of *Cannabis sativa* L. cultivars (F—Finola, FR—Futura 75, D—Dioica, K—Kompolti). Data are presented as mean values, different letters above the bars indicate significant differences among the analyzed groups (Tukey's HSD test, $p < 0.000$).

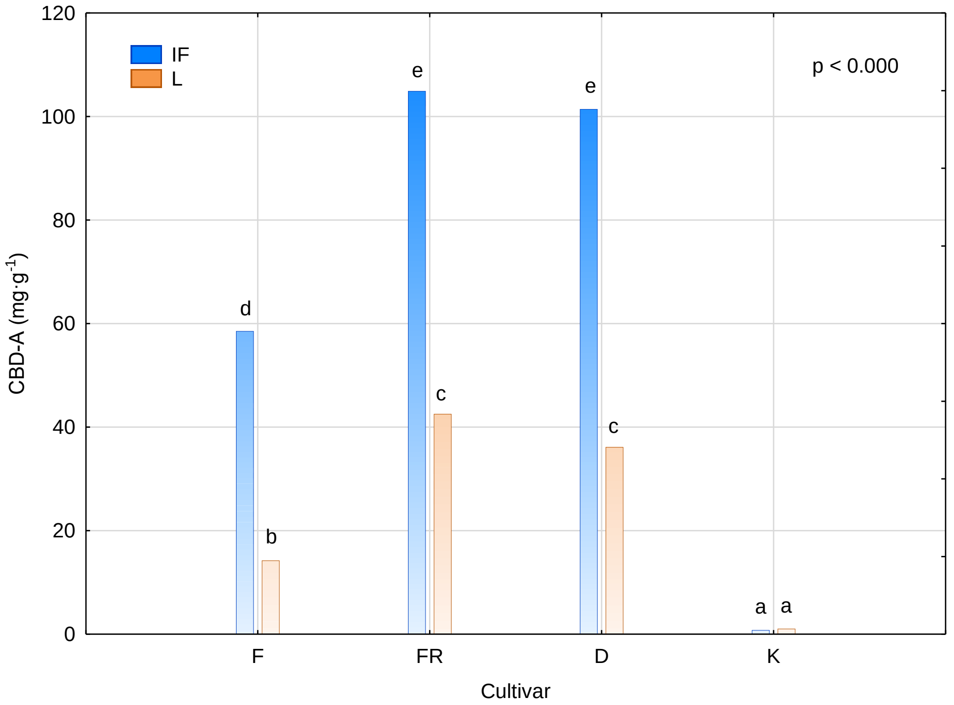

Figure S5. CBD-A content in the leaves (L) and inflorescences (IF) of *Cannabis sativa* L. cultivars (F—Finola, FR—Futura 75, D—Dioica, K—Kompolti). Data are presented as mean values, different letters above the bars indicate significant differences among the analyzed groups (Tukey's HSD test, $p < 0.000$).

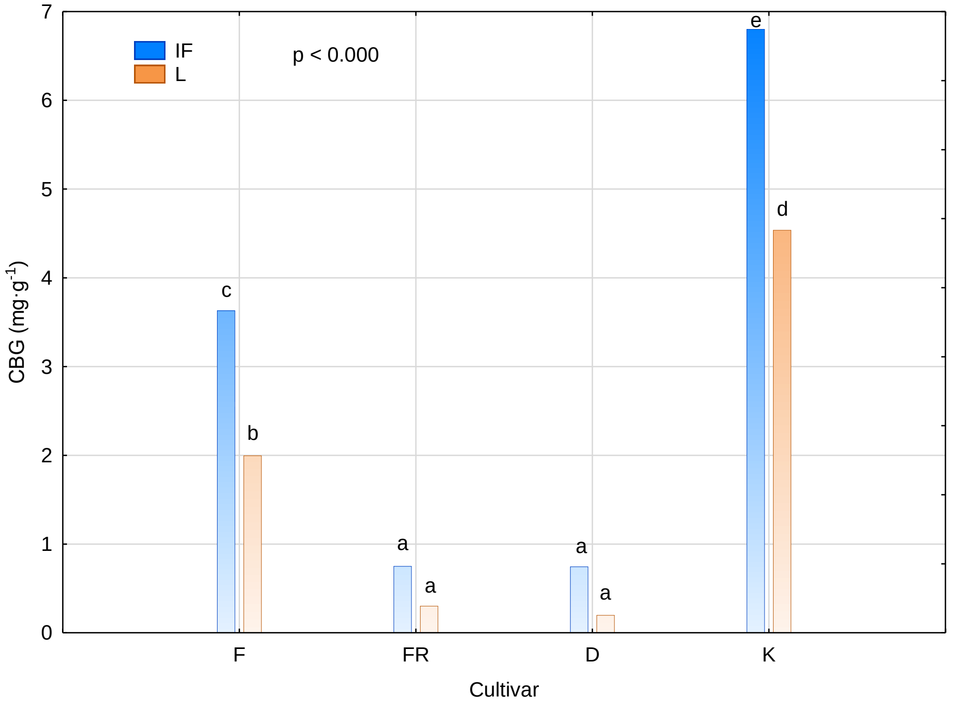

Figure S6. CBG content in the leaves (L) and inflorescences (IF) of *Cannabis sativa* L. cultivars (F—Finola, FR—Futura 75, D—Dioica, K—Kompolti). Data are presented as mean values, different letters above the bars indicate significant differences among the analyzed groups (Tukey's HSD test, $p < 0.000$).

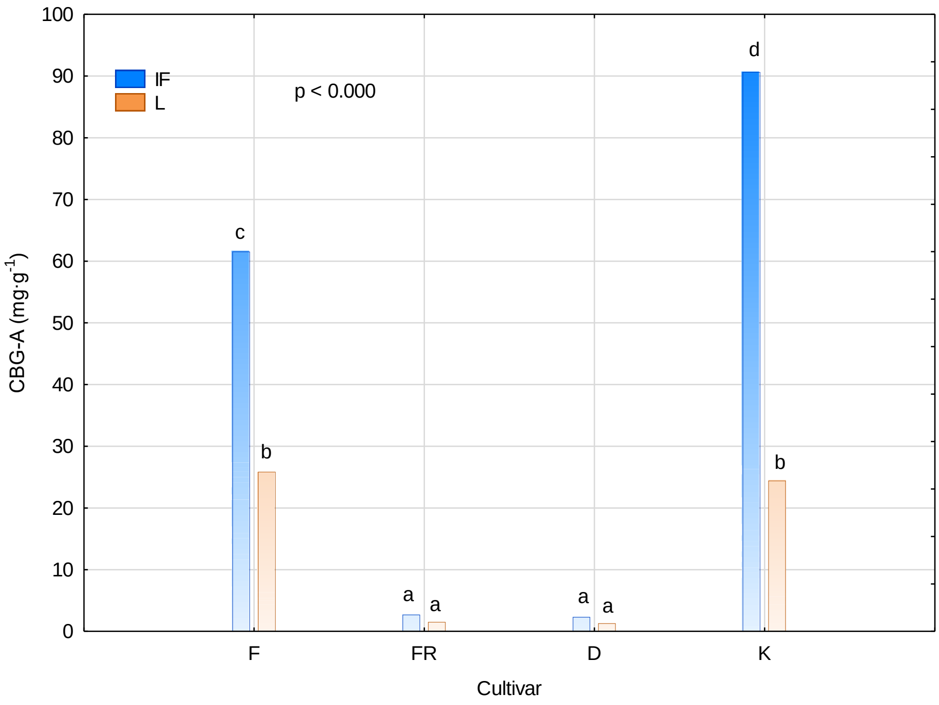

Figure S7. CBG-A content in the leaves (L) and inflorescences (IF) of *Cannabis sativa* L. cultivars (F—Finola, FR—Futura 75, D—Dioica, K—Kompolti). Data are presented as mean values, different letters above the bars indicate significant differences among the analyzed groups (Tukey's HSD test, $p < 0.000$).

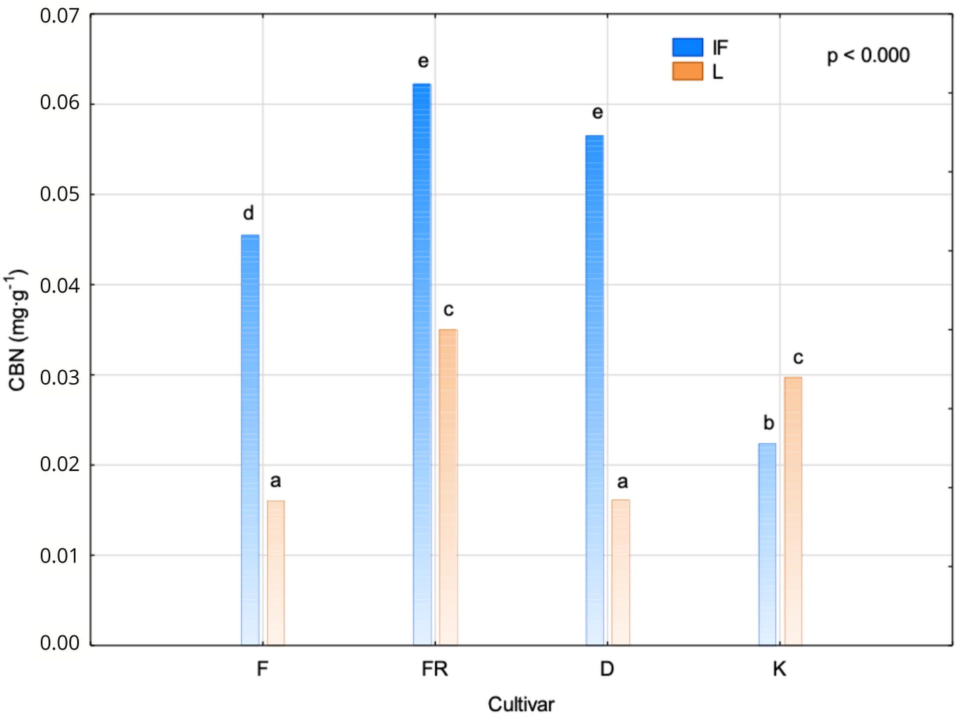

Figure S8. CBN content in the leaves (L) and inflorescences (IF) of *Cannabis sativa* L. cultivars (F—Finola, FR—Futura 75, D—Dioica, K—Kompolti). Data are presented as mean values, different letters above the bars indicate significant differences among the analyzed groups (Tukey's HSD test, $p < 0.000$).

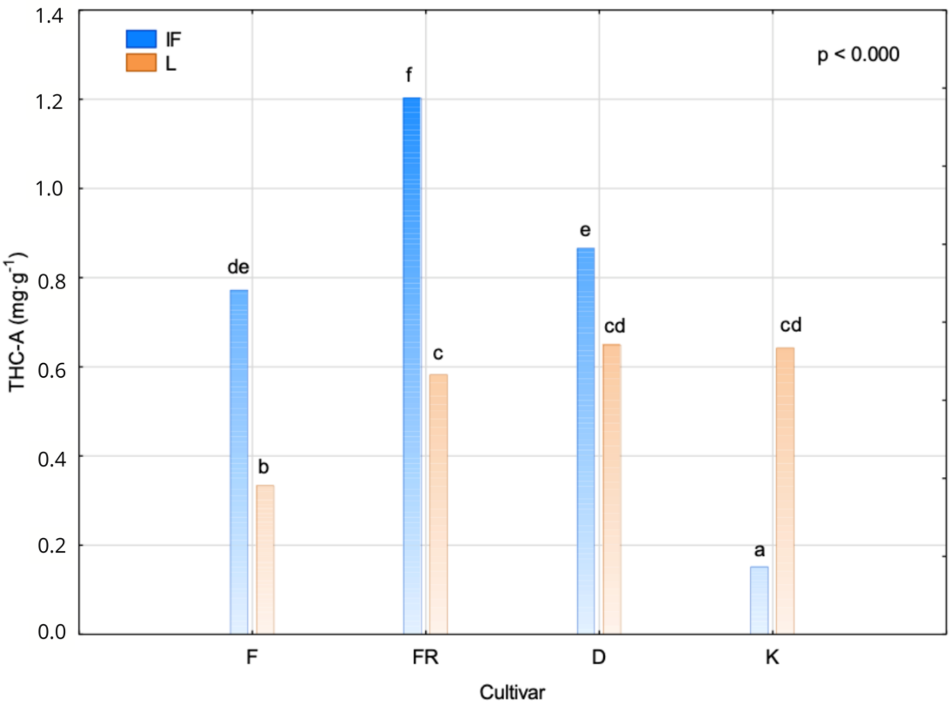

Figure S9. THC-A content in the leaves (L) and inflorescences (IF) of *Cannabis sativa* L. cultivars (F—Finola, FR—Futura 75, D—Dioica, K—Kompolti). Data are presented as mean values, different letters above the bars indicate significant differences among the analyzed groups (Tukey's HSD test, $p < 0.000$).

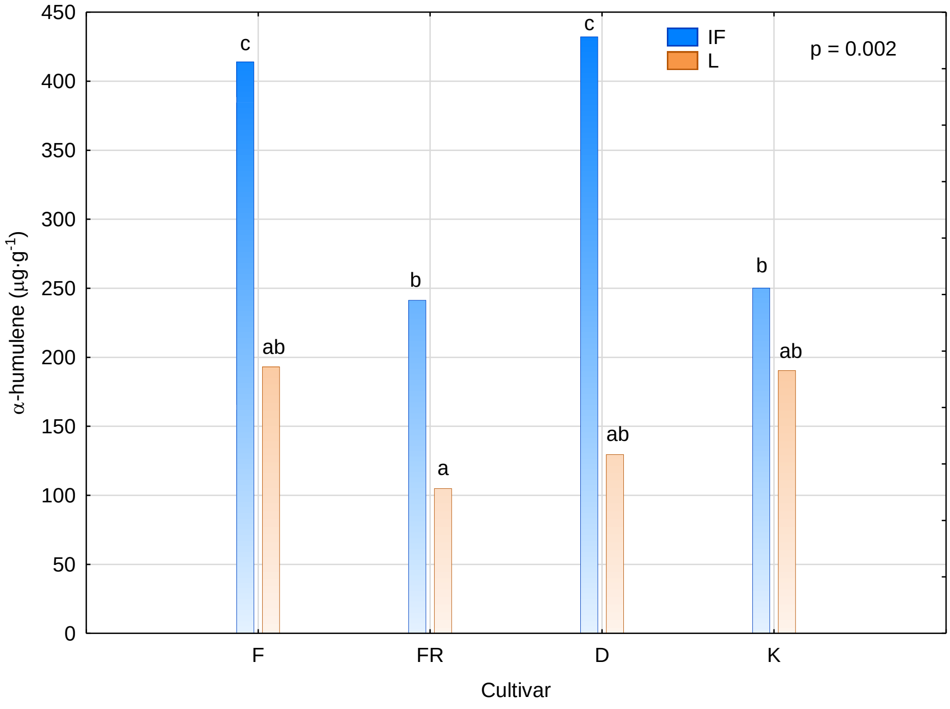

Figure S10. α-humulene content in the leaves (L) and inflorescences (IF) of *Cannabis sativa* L. cultivars (F—Finola, FR—Futura 75, D—Dioica, K—Kompolti). Data are presented as mean values, different letters above the bars indicate significant differences among the analyzed groups (Tukey's HSD test, $p = 0.002$).

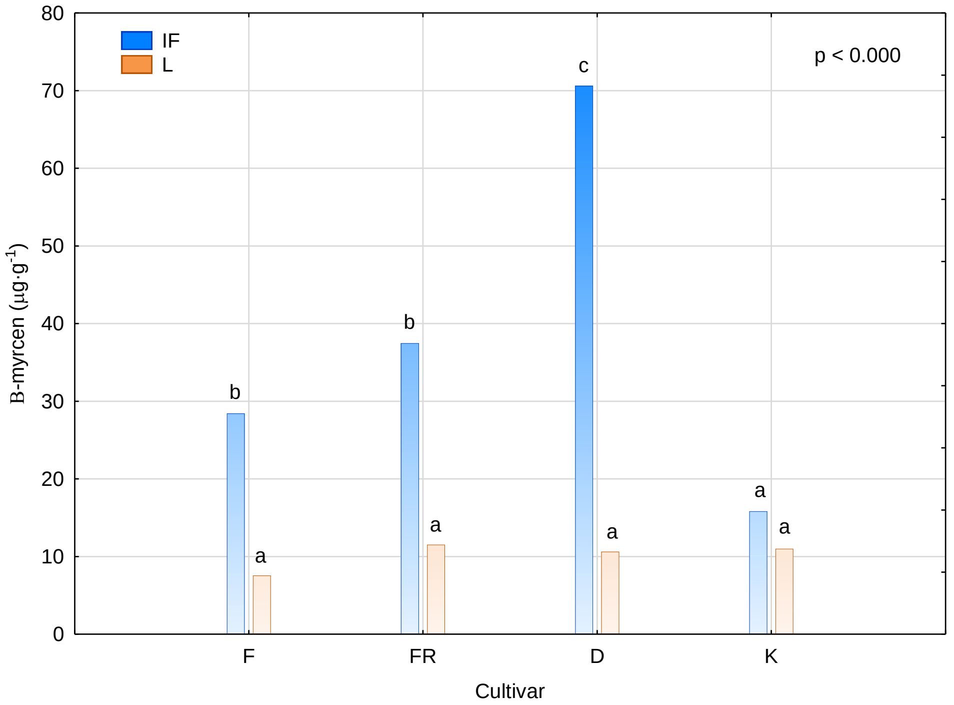

Figure S11. β-myrcene content in the leaves (L) and inflorescences (IF) of *Cannabis sativa* L. cultivars (F—Finola, FR—Futura 75, D—Dioica, K—Kompolti). Data are presented as mean values, different letters above the bars indicate significant differences among the analyzed groups (Tukey's HSD test, $p < 0.000$).

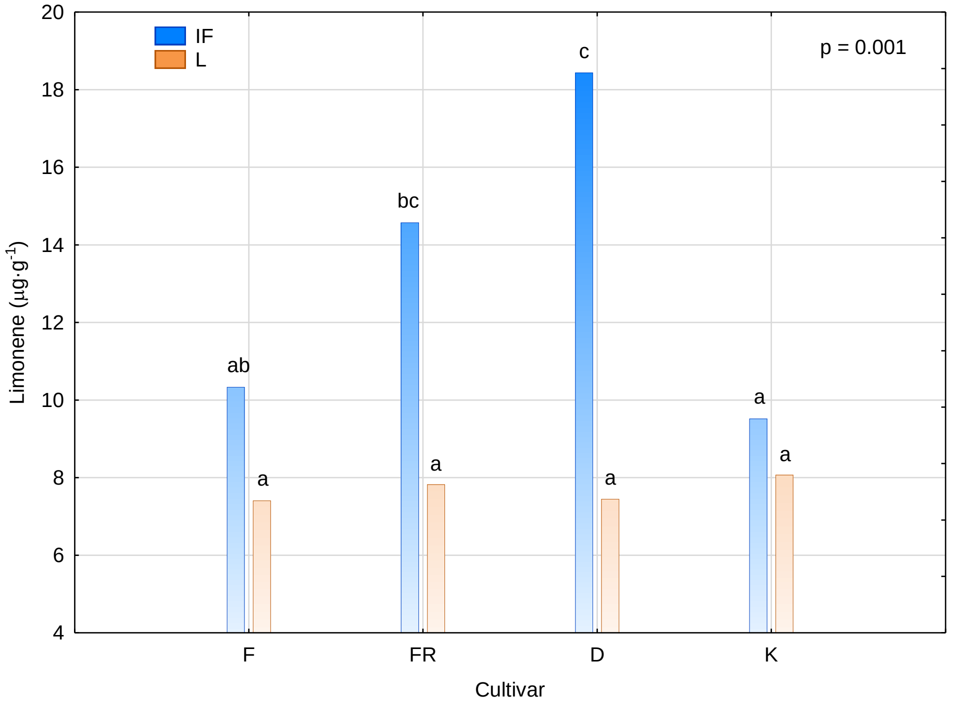

Figure S12. Limonene content in the leaves (L) and inflorescences (IF) of *Cannabis sativa* L. cultivars (F—Finola, FR—Futura 75, D—Dioica, K—Kompolti). Data are presented as mean values, different letters above the bars indicate significant differences among the analyzed groups (Tukey's HSD test, $p = 0.001$).

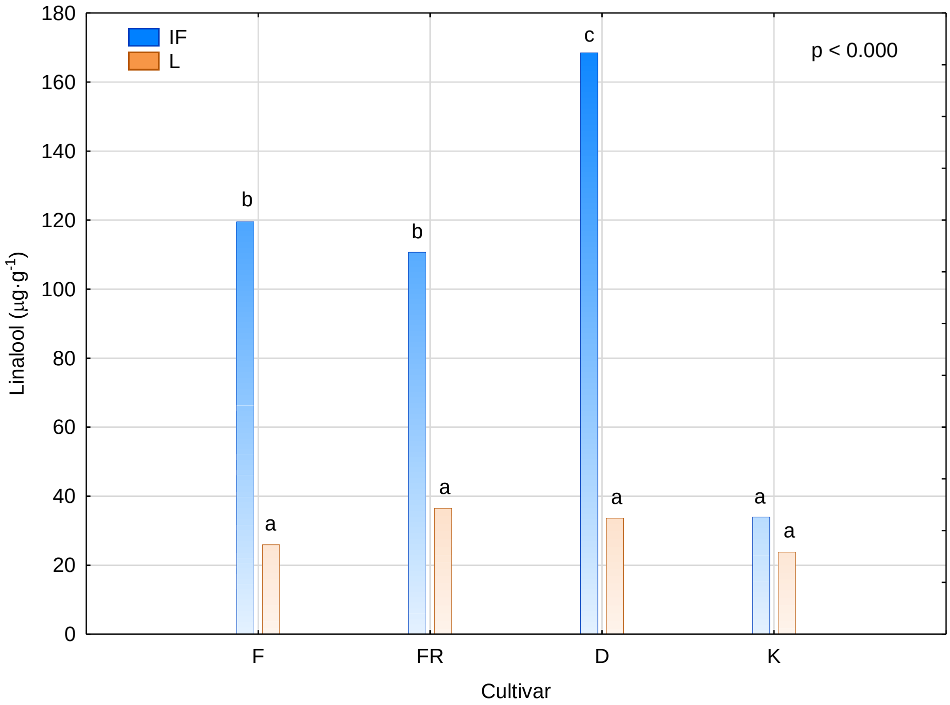

Figure S13. Linalool content in the leaves (L) and inflorescences (IF) of *Cannabis sativa* L. cultivars (F—Finola, FR—Futura 75, D—Dioica, K—Kompolti). Data are presented as mean values, different letters above the bars indicate significant differences among the analyzed groups (Tukey's HSD test, $p < 0.000$).

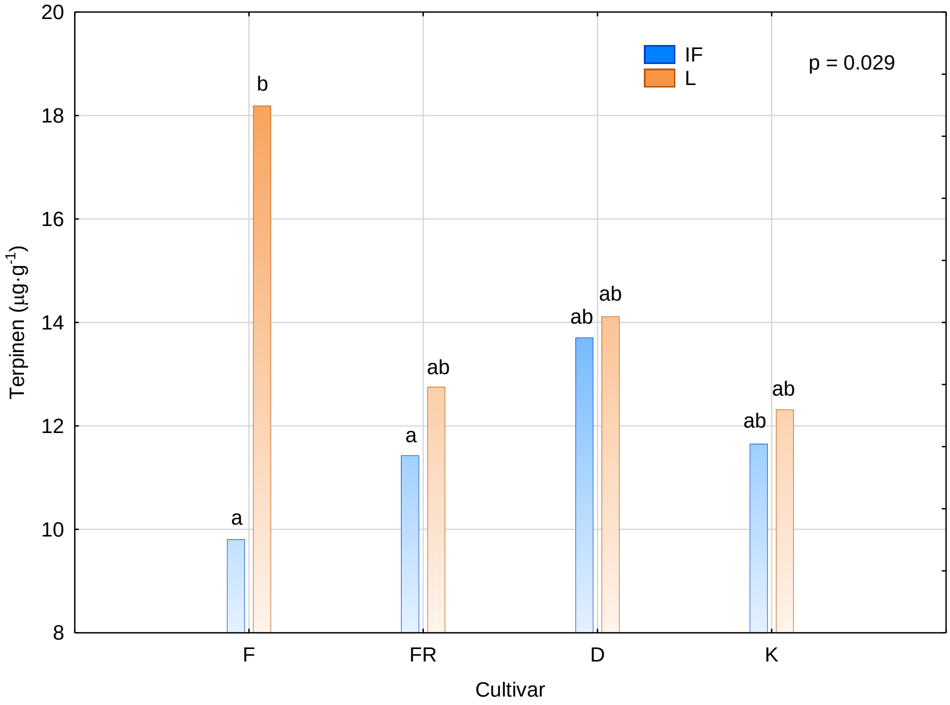

Figure S14. Terpinen content in the leaves (L) and inflorescences (IF) of *Cannabis sativa* L. cultivars (F—Finola, FR—Futura 75, D—Dioica, K—Kompolti). Data are presented as mean values, different letters above the bars indicate significant differences among the analyzed groups (Tukey's HSD test, $p = 0.029$).
